# PdCu nanoalloy decorated photocatalysts for efficient and selective oxidative coupling of methane in flow reactors

**DOI:** 10.1038/s41467-023-41996-y

**Published:** 2023-10-10

**Authors:** Xiyi Li, Chao Wang, Jianlong Yang, Youxun Xu, Yi Yang, Jiaguo Yu, Juan J. Delgado, Natalia Martsinovich, Xiao Sun, Xu-Sheng Zheng, Weixin Huang, Junwang Tang

**Affiliations:** 1 Department of Chemical Engineering, University College London, London, WC1E 7JE UK; 2 Key Lab of Synthetic and Natural Functional Molecule Chemistry of Ministry of Education, the Energy and Catalysis Hub, College of Chemistry and Materials Science, Northwest University, Xi’an, P. R. China; 3 State Key Laboratory of Advanced Technology for Materials Synthesis and Processing, Wuhan University of Technology, Wuhan, 430070 China; 4 Laboratory of Solar Fuel, Faculty of Materials Science and Chemistry, China University of Geosciences, 388 Lumo Road, Wuhan, 430074 China; 5 Departamento de Ciencia de los Materiales e Ingeniería Metalúrgica y Química Inorgánica, Facultad de Ciencias, Universidad de Cádiz, Campus Rio San Pedro, 11510 Puerto Real, Cádiz Spain; 6 IMEYMAT, Instituto de Microscopía Electrónica y Materiales, Puerto Real, 11510 Spain; 7 Department of Chemistry, University of Sheffield, Sheffield, S3 7HF UK; 8 Hefei National Research Center for Physical Sciences at the Microscale, iChEM, Key Laboratory of Surface and Interface Chemistry and Energy Catalysis of Anhui Higher Education Institutes, School of Chemistry and Materials Science, University of Science and Technology of China, 230026 Hefei, China; 9 National Synchrotron Radiation Laboratory, University of Science and Technology of China, Hefei, 230029 Anhui China; 10 Industrial Catalysis Center, Department of Chemical Engineering, Tsinghua University, Beijing, 100084 China

**Keywords:** Photocatalysis, Photocatalysis, Inorganic chemistry

## Abstract

Methane activation by photocatalysis is one of the promising sustainable technologies for chemical synthesis. However, the current efficiency and stability of the process are moderate. Herein, a PdCu nanoalloy (~2.3 nm) was decorated on TiO_2_, which works for the efficient, stable, and selective photocatalytic oxidative coupling of methane at room temperature. A high methane conversion rate of 2480 μmol g^−1^ h^−1^ to C_2_ with an apparent quantum efficiency of ~8.4% has been achieved. More importantly, the photocatalyst exhibits the turnover frequency and turnover number of 116 h^−1^ and 12,642 with respect to PdCu, representing a record among all the photocatalytic processes (λ > 300 nm) operated at room temperature, together with a long stability of over 112 hours. The nanoalloy works as a hole acceptor, in which Pd softens and weakens C-H bond in methane and Cu decreases the adsorption energy of C_2_ products, leading to the high efficiency and long-time stability.

## Abstract (web summary)

Efficient and stable photocatalytic oxidative coupling of methane to C2 products is revealed by Tang’s group via synergistic effects of a PdCu nanoalloy cocatalyst which achieves high TON_PdCu_ of 12642 and TOF_PdCu_ of 116 h^−1^ with >100 hour stability.

## Introduction

The discovery of fire ice, together with the shale gas has indicated that methane reserve on the earth is much more than the sum of coal and oil, which renders methane as one of the most promising future energy sources and chemical feedstocks^1,2^. However, due to the inert nature of methane (high symmetrical structure, low polarisability, high ionization potential, and highly stable C-H bond), the activation energy for methane conversion is high, resulting in harsh reaction conditions, such as high temperatures, strong oxidants, or strong acidic environments^3–5^. Therefore, the direct conversion of methane via traditional processes has suffered from several barriers to commercialisation, including the deactivation of catalysts (e.g., coke accumulation and sintering), low selectivity of desired products (e.g., overoxidation), large CO_2_ emission and high capital costs^6–8^. Furthermore, this does not align with the goals of transition to clean technology with net zero carbon emissions as underscored by the UN Climate Change Conference UK 2021 (COP 26).

Photocatalysis, a developing green technology, utilises photon-induced charge carriers (electrons/holes) to pre-activate stable chemical bonds (e.g., O-H, C = O, and C-H), thus lowering activation barriers and driving thermodynamically unfavourable chemical reactions (water splitting, CO_2_ reduction and methane conversion, etc.) under mild conditions^7,9,10^. Among various methane conversion reactions, coupling of two methane molecules to produce C_2_ products (C_2_H_6_/C_2_H_4_) is one of the most general and highly profitable processes^3,11^, while it is much more challenging than C_1_ production. We have recently reported the first example of photocatalytic oxidative coupling of methane (OCM) over CuO_x_-Pt/TiO_2_ in a flow reactor^12^, which proved the concept while the C_2_ yield rate was moderate (6.8 μmol h^−1^) and the stability test only ran for 8 h.

Photocatalytic processes often exhibit high selectivity, however, the reported conversion rate and more importantly the stability are rather moderate, which are two equally important indexes, especially for future application^2^. Two important factors, Turnover frequency (TOF) and Turnover number (TON), reflecting both catalytic activity and stability, are robust to assess the photocatalysts. The majority of the reported photocatalytic methane coupling flow systems represent a rather moderate TOF < 5 h^−1^ or TON of <10. Very recently a few benchmark studies such as oxidative coupling with water over Pd/Ga_2_O_3_^13^, non-oxidative coupling over Au/TiO_2_^14^_,_ and oxidative coupling with oxygen over Au-ZnO/TiO_2_^15^, improved TON to a few hundred, with the assistance of deep UV light (254 nm)^13^ or the thermal effect (140 °C)^15^. It is still a great challenge to achieve an efficient photocatalyst with both high TOF and TON for photocatalytic coupling of methane under ambient conditions.

A novel design strategy of multi-functional photocatalysts is necessary to achieve this goal, which requires the synergy of different components. Formation of bimetallic nanoalloy catalysts via combining one metal with another is one of the most effective and universal strategies. Successful examples have been reported in traditional methane conversion to obtain both high catalytic performance and resistance to deactivation, such as steam reforming of methane over AuNi^16^ and dry reforming of methane over Ni-Cu/Mg(Al)O^17^. In photocatalytic methane conversion, three factors need to be considered to meet this versatile requirement, such as (i) high availability of photon-induced charge carriers; (ii) efficient and selective activation of the first C-H bond in CH_4_; (iii) prolonged stability against coking and bleaching of active species.

Herein, a screening process was carried out assess a series of noble metals on the TiO_2_ photocatalyst, which usually act as charge sinks to promote charge transfer in photocatalysis^18^ and show unique catalytic performance for the C-H bond activation in thermocatalysis^19^. Pd shows exceptional activity as the most efficient co-catalyst but suffers from coke accumulation like a recent example in photocatalytic methane conversion over Pd/Ga_2_O_3_^20^. In traditional methane conversion, the coke resilience could be enhanced by the formation of nanoalloy with another active transition metal, e.g., PtCu, NiCu^21,22^. Thus, a series of non-noble transition metals as the second component were introduced to form bimetallic nanoalloys with Pd to enhance the stability. The incorporation of Cu is found to enhance both activity and resistance to coking of the photocatalyst. The PdCu nanoalloy decorated TiO_2_ exhibits the highest methane conversion rate of 2480 μmol g^−1^ h^−1^ to C_2_ products with the high quantum efficiency (AQE) of ~8.4% at 365 nm. Furthermore, PdCu/TiO_2_ presents a TOF_PdCu_ of 116 h^−1^ and TON_PdCu_ of 12,642 for the conversion to C_2_ products, which is 20 times higher than the recent benchmark results^14,23^.

## Results

### Photocatalytic activity

**Fig. 1:**
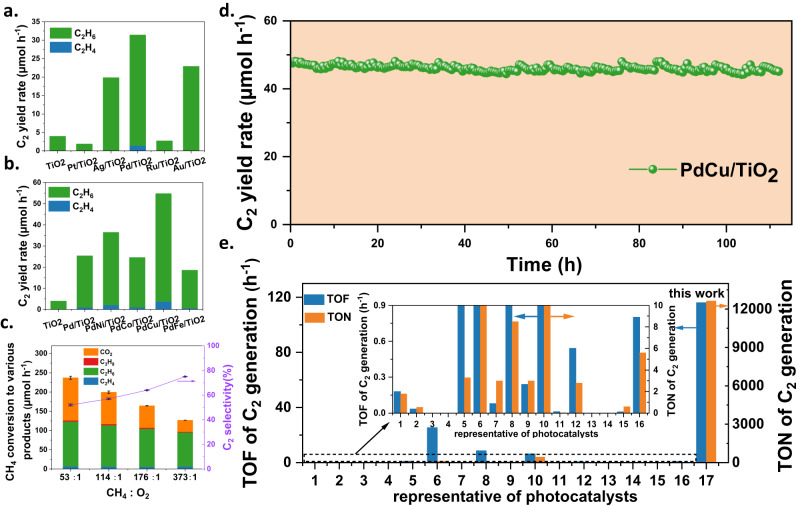
Photocatalytic OCM activity over PdCu nanoalloy decorated TiO_2_. **a** C_2_ yield rate over different noble co-catalysts decorated TiO_2_ with the equal molar amount to Pd. **b** Influence of second transition metal cocatalysts with the equal molar amount to Pd on C_2_ production at 1.5-hour run. **c** CH_4_ total conversion rate and C_2_ selectivity over PdCu/TiO_2_ with different CH_4_: O_2_ ratios (the ratios were 53: 1, 114: 1, 176: 1, and 373: 1, respectively). Error bars (standard deviation) in the figure were obtained from three sampling runs. **d** C_2_ synthesis over PdCu/TiO_2_ during an intermittent 112-hour reaction with the CH_4_: O_2_ ratio of 373: 1, these data were collected every 30 min. **e** Summary of turnover frequency (TOF) and turnover number (TON) achieved on PdCu/TiO_2_ and other reported representative photocatalysts (λ > 300 nm) at room temperature (1: AuPd/ZnO^79^; 2: Au/ZnO^80^; 3: Ga_2_O_3_-K^81^; 4: MgO-SiO_2_^82^; 5: Ce-Al_2_O_3_^83^; 6: Au/WO_3_^23^; 7: Ag-H_3_PW_12_O_40_/TiO_2_^33^; 8: Pt-CuO_x_/TiO_2_^12^; 9: Pt/Ga-TiO_2_-SiO_2_^84^; 10: Au/TiO_2_^14^; 11: TiO_2_/SiO_2_^85^; 12: SiO_2_-Al_2_O_3_-TiO_2_^86^; 13: H-MOR^87^; 14: FSM-16^88^; 15: GaN: ZnO^89^; 16: Zn_5_(OH)_8_Cl_2_·H_2_O^90^, 17: this work.). The insert figure is the zoomed rectangle region indicated with the dash line in the main panel, showing the range of TON between 0–10 and TOF between 0–0.9. Among them, the values of photocatalyst 3, 4, 13 and 14 are still too small to see even in the enlarged insert. The calculation method is shown in the Method section. Reaction condition in (**a**, **b**): gas hourly space velocity (GHSV = 342,000 mL g_cat_^−1^ hour^−1^), CH_4_: O_2_ = 114: 1, 10% of CH_4_, Ar as balance gas, 365 nm LED 40 W, 30 °C; the reaction condition in (**c**) was similar to the above except the ratio of CH_4_: O_2_ and the reaction condition in (**d**) was also similar except the ratio of CH_4_: O_2_ was changed to 373: 1. Source data are provided as a Source Data file.

Since noble metals are often regarded as charge sinks to promote charge separation to facilitate photocatalytic activity^18^, photocatalytic OCM reactions were first investigated over different noble metals nanoparticles decorated TiO_2_ (anatase) in a flow reactor using a 365 nm LED under room temperature and atmospheric pressure (Fig. 1a). Different effects on the C_2_ yield rates were observed, following the trend: Pd/TiO_2_ > Au/TiO_2_ > Ag/TiO_2_ > TiO_2_ > Ru/TiO_2_ > Pt/TiO_2_. Compared with the low yield rate (ca. 4 μmol h^−1^) of pristine TiO_2_, an improvement of nearly 8 times is achieved after the loading of Pd. In addition to the enhancement of charge carrier transfer, such a promotion effect can also be attributed to the strong interaction between Pd nanoparticles and the C-H bond in CH_4_^24,25^. Pd was then selected as the best co-catalyst and its loading amount was optimized (Supplementary Fig. 1). The C_2_ yield rate exhibited a volcano trend with the increasing loading wt% of Pd. After reaching 32 μmol h^−1^ over Pd_0.1_/TiO_2_, less than 20% increase could be observed with a five-fold increase in Pd loading amount, and further increase of loading amount to 1 wt% resulted in decreased activity. This might be because excessive Pd nanoparticles obstruct the light penetration to TiO_2_ by scattering and/or absorption^26,27^. Balancing the cost and performance of Pd cocatalyst, 0.1 wt% was selected as the optimum amount for the following study (denoted Pd/TiO_2_ without specific notification). Unexpectedly, the performance of Pd/TiO_2_ exhibited a declined trend when the reaction time was extended, as shown in Supplementary Fig. 2. In thermocatalytic methane conversion, the strong interaction of the catalyst with CH_4_ (e.g., strong binding force to adsorbates or low activation energy barrier) often lead to consecutive reactions for coke accumulation^19,28^. A recent example in photocatalytic methane conversion over Pd/Ga_2_O_3_ also found the deposition of coke by a side reaction^20^. Thus, the deactivation of Pd/TiO_2_ is likely due to the accumulation of cokes, which is proved later in this study.

Transition metals can be used to form alloys with another active metal to enhance the cokes resilience^21,22^. Thus, a group of transition metals were investigated to form nanoalloy with Pd and their molar amount was equal to that of Pd. Figure 1b shows that Cu and Ni both improve the Pd/TiO_2_ activity for C_2_ yield among Ni, Co, Cu, and Fe cocatalysts loaded photocatalysts, nearly 2.2 and 1.4 times higher than single Pd/TiO_2_, respectively.

More interestingly, PdNi/TiO_2_, PdCo/TiO_2_, and PdFe/TiO_2_, exhibit a declined trend similar to Pd/TiO_2_ for C_2_ yield rates, while the incorporation of Cu reverses the trend of C_2_ yield rate (Supplementary Fig. 2), so the PdCu is the best.. The effect of the Pd: Cu ratio and the PdCu loading amount was also optimised as discussed alongside in Supplementary Fig. 3 and 4. The optimum sample is Pd_0.1_Cu_0.1_/TiO_2_ and the real concentration of the cocatalyst was then analysed by ICP-OES to be Pd 0.089% and Cu 0.051% listed in Supplementary Table 1 (denoted PdCu/TiO_2_ unless otherwise specified). Supplementary Fig. 5 further shows temporal C_2_ yield on different photocatalysts. Both Cu/TiO_2_ and PdCu/TiO_2_ show an almost stable activity while Pd/TiO_2_ activity keeps a fast decay and then a slow decay with time. The yield rate of C_2_ on Pd/TiO_2_ decreases by 50% when the reaction time is prolonged to 5 h. PdCu/TiO_2_ represents the stable yield rate of ca. 57 μmol h^−1^ for 8 h. Supplementary Fig. 6 details the selectivity to C_2_ products, CO_2_, and C_3_H_8_. The introduction of Cu, Pd, and PdCu increases C_2_ selectivity to 37%, 55%, and 57% compared with the pristine TiO_2_ (13%), respectively, and the total methane conversion rate on PdCu/TiO_2_ is more than 3 times higher than that of pristine TiO_2_. Afterward, the ratio of CH_4_ to O_2_ was varied to investigate the optimum reaction conditions (Fig. 1c). When the ratio of CH_4_ to O_2_ is 53: 1, the C_2_ yield rate reaches as high as 62 μmol h^−1^ (1240 μmol g^−1^ h^−1^), corresponding to the methane conversion to C_2_ rate of 124 μmol h^−1^ (2480 μmol g^−1^ h^−1^), showing the leading position in the reported photocatalytic methane conversion to C_2_ products processes under ambient conditions (Supplementary Table 2). The optimised catalyst also exhibits a high light utilisation efficiency with a apparent quantum efficiency (AQE) of ~8.4% at 365 nm. Excessive amounts of O_2_ would generate more superoxide radicals O_2_·^-^, which was considered as an active species to promote the conversion rate but also to increase overoxidation to CO_2_^27,29^. Thus, as the O_2_ amount is reduced (with the CH_4_: O_2_ ratio increased from 53: 1 to 373: 1), a slight decrease in methane conversion rate to ethane is observed. By contrast, the overoxidation to CO_2_ shows a notable decrease to less than one third, from ca. 110 to 30 μmol h^−1^, resulting in a high C_2_ selectivity of ca. 75%. In addition, such high selectivity is comparable to the most excellent OCM catalysts (e.g., Li/MgO; Mn/Na_2_WO_4_/SiO_2_) by thermocatalysis operated at >873 K^30,31^. Therefore, the optimised experimental condition is the gas hourly space velocity (GHSV) of 342,000 mL g_cat_^−1^ hour^−1^ with the ratio of CH_4_ to O_2_ at 373: 1 operated at room temperature.

The control experiments were also carried out and they show that no products are observed in the absence of methane, without photocatalysts or under dark conditions, proving that this is a photocatalytic process and the only carbon source is methane (Supplementary Fig. 7). To further confirm methane as the only carbon source^13^,CH_4_ isotope experiment was carried out (Supplementary Fig. 8). Dominant peaks attributed to main products and their molecular fragments can be observed using ^12^CH_4_ as the feedstock, such as C_2_H_6_ (m/z = 30, 29, 28, 27, 26), C_2_H_4_ (m/z = 28, 27, 26) and CO_2_ (m/z = 44, 28), respectively. When the ^13^C-labelled CH_4_ is used as the feedstock, the m/z of dominant peaks increase by 2 for both ^13^C_2_H_6_ (32, 31, 30, 29, 28) and ^13^C_2_H_4_ (m/z = 30, 29, 28). In parallel, the m/z of ^13^CO_2_ (m/z = 45, 29) also shifts by 1 compared with that observed under the ^12^CH_4_ atmosphere. Although some m/z for product ^13^C_2_H_6_ may overlap with other components (e.g., m/z of 32 for ^13^C_2_H_6_ and O_2_, m/z of 30 for ^13^C_2_H_4_· and ^12^C_2_H_6_), the intensity of these m/z is much higher than that using ^12^CH_4_ as the feedstock. This result further indicates that the large contribution for the intensity at m/z 32 and m/z 30 should come from the ^13^C isotope-labelling products. More importantly, the m/z of 31 indicating the formation of ^13^C_2_H_5_· can be observed under the ^13^CH_4_ atmosphere. The disappearance of m/z = 26 and 27 for molecular fragments of ^12^C_2_H_2_· and ^12^C_2_H_3_· further confirms the carbon source from ^13^CH_4_.

Then, a long-term stability test was carried out under the optimised condition as shown in Fig. 1d. The CH_4_ conversion rate to C_2_ remains almost stable during the whole term of the 112-hour test despite a slight change likely due to slight fluctuation of the gas flow rates. This result indicates the high stability of PdCu nanoalloy as the catalytic active site. This is one of the longest times (>100 h) for stability tests in photocatalytic methane conversion, although it is not uncommon in thermocatalysis to evaluate a catalyst with such a long reaction time^6,32^. TOF and TON, reflecting the efficiency and stability of a catalyst, were used to assess the PdCu nanoalloy co-catalyst against other photocatalysts, as shown in Fig. 1e (the detailed calculation process of TOF and TON was shown in Methods). The reported representative photocatalysts in methane conversion to C_2_ products generally exhibit a very small TON of <10 and a moderate TOF of <5 h^−1^, and two very recent benchmark work achieved TON of 442 h ^14^and TOF of 25 h^−1 23^, respectively. PdCu/TiO_2_ shows the highest turnover frequency (TOF_PdCu_) of C_2_ generation (116 h^−1^) among all the reported photocatalysts operated at λ > 300 nm and room temperature, being a new benchmark to the best of our knowledge. Equally importantly, the TON_PdCu_ under the optimised condition is as high as 12,642. This result indicates that PdCu nanoalloy is a stable and effective active site for the selective conversion of methane to C_2_ products.

### Photocatalysts characterisation

**Fig. 2:**
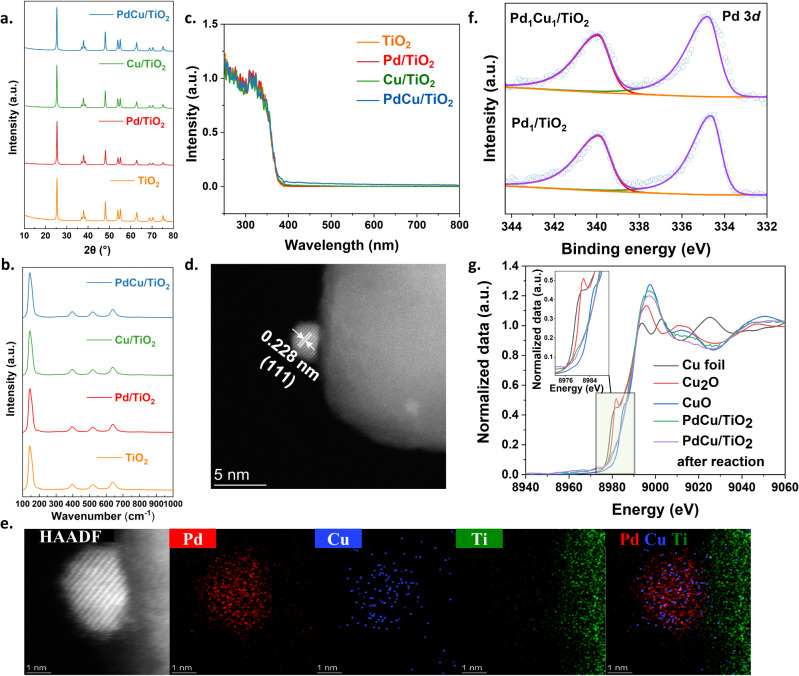
Characterisation of the photocatalysts. **a** XRD spectra of TiO_2_, Pd/TiO_2_, Cu/TiO_2_ and PdCu/TiO_2_. **b** Raman spectra of TiO_2_, Pd/TiO_2_, Cu/TiO_2_ and PdCu/TiO_2_. **c** UV-Vis absorption spectra of TiO_2_, Pd/TiO_2_, Cu/TiO_2_ and PdCu/TiO_2_. **d** HR-TEM image of PdCu/TiO_2_. **e** HAADF-STEM image and EDX elements mapping of PdCu/TiO_2_. **f** XPS spectra of Pd_1_/TiO_2_ and Pd_1_Cu_1_/TiO_2_ (Pd 3*d*) before reaction. **g** Cu K-edge XANES spectra of PdCu/TiO_2_ and PdCu/TiO_2_ after reaction in reference to Cu foil, Cu_2_O and CuO. Source data are provided as a Source Data file.

The structure of the photocatalysts was first investigated by powder X-ray diffraction (PXRD), as shown in Fig. 2a. All samples exhibit the typical peaks assigned to the anatase phase (JCPDS no. 84-1286). Neither Pd nor Cu diffraction peaks are observed in all modified samples, probably owing to the low content (Supplementary Table 2) or high dispersion^33^. Raman spectra were further used to investigate the structure (Fig. 2b). Consistent with the results of PXRD, all samples display typical Raman peaks of the anatase phase, at 144 cm^−1^(*E*_g_), 198 cm^−1^(*E*_g_), 399 cm^−1^(*B*_1g_), 512 cm^−1^(*A*_1g_), and 639 cm^−1^(*E*_g_), respectively, suggesting the stable framework of anatase TiO_2_ after cocatalyst loading. The ultraviolet-visible diffuse reflectance spectroscopy (UV-Vis DRS) was used to study the photon-absorption properties (Fig. 2c). The absorption (both absorption peak and absorption edge) of all samples remains almost the same, indicating that TiO_2_ harvests light for photon-induced carrier generation. A slightly enhanced light absorption at the visible light region after the introduction of co-catalyst nanoparticles is observed, probably due to the interband absorption and/or scattering by these particles^34^.

All technologies mentioned above could not provide effective information on the small PdCu nanoalloy particles, thus high-angle annular dark-field scanning transmission electron microscopy (HAADF-STEM) was carried out to gain a microscale view of the PdCu/TiO_2_ sample (Supplementary Fig. 9). It should be noted that bright nanoparticles are distributed evenly on the supports, with an average size of 2.3 nm. These nanoparticles were further identified by the high-resolution TEM (HR-TEM) image (Fig. 2d), in which the distinct lattice fringes are seen with a d-spacing of 0.228 nm, corresponding to the (111) plane of PdCu nanoalloy^35,36^. To confirm the formation of PdCu nanoalloy, the Energy Dispersive X-Ray Analysis (EDX) was applied to scan the nanoparticles (Fig. 2e, Supplementary Fig. 10). Both Pd and Cu are distributed homogenously within the 2 nm nanoparticle, and a distinct boundary could be identified to distinguish the nanoparticle from TiO_2_. Moreover, the element intensity for Pd or Cu can only be visible within the nanoparticle and no signal of individual Pd or Cu can be detected on the TiO_2_ support by the STEM image (Supplementary Fig. 10), confirming the accuracy of the element mapping for PdCu detection. The PdCu nanoalloy structure and the average size remain unchanged after 8 h of reaction (Supplementary Fig. 11–12), indicating the high stability of these nanoparticles.

X-ray photoelectron spectroscopy (XPS) was then carried out to analyse the chemical states of the co-catalyst. Pd_1_/TiO_2_ and Pd_1_Cu_1_/TiO_2_ with larger loading amounts (1 wt%) of co-catalysts were used in order to have a higher resolution of the spectra. Both samples exhibit similar C_2_ yield trend with increasing time, like their counterparts with low loading amount (Supplementary Fig. 13). The Pd 3*d* XPS spectra of Pd_1_Cu_1_/TiO_2_ exhibited two peaks at 334.7 eV Pd 3*d*_5/2_ and 340.0 eV Pd 3*d*_3/2_, respectively (Fig. 2f), and Pd_1_/TiO_2_ showed similar peaks at 334.6 eV Pd 3*d*_5/2_ and 339.9 eV Pd 3*d*_3/2_, respectively. Both of them were identical to the metallic states of Pd, and the small positive shift of 0.1 eV after the introduction of Cu can be attributed to the interaction between Pd and Cu^37^. No change of Pd 3*d* spectra after 6-hour photocatalytic OCM reaction can be observed, suggesting the high stability of Pd species (Supplementary Fig. 14).

Since the peak positions for Cu/Cu^I^/Cu^II^ are too closed and the satellite peak for Cu^II^ may be neglected as the background in our case (Supplementary Fig. 15), the Wagner plot was used to analyse the chemical state of Cu species (detailed discussion along with Supplementary Fig. 16 and Supplementary Table 3), which shows the highly possible existence of Cu(II) species in PdCu/TiO_2_. To further identify the presence of Cu(0) or Cu(I) species, the XAS was carried out. At the Cu K-edge (Fig. 2g), the positive shift in the absorption edge suggests the partial oxidation of Cu species in the PdCu nanoalloy^38^. One can see that the feature of PdCu/TiO_2_ exhibits a great difference from that of Cu_2_O, indicating that CuO is the main oxidised species in the sample. The degree of Cu oxidation relies on the exposure time in air and a longer time may lead to more surface Cu oxidation. This is common in PdCu nanoalloy particles according to previous reports, since the Cu on the surface of PdCu nanoalloy can be oxidised more easily^39,40^. To gain a deeper understanding of the local structure of the alloy nanoparticle, the Fourier transformation magnitudes of k^3^−weighted EXAFS data and theoretical fitting for Cu K-edge of PdCu/TiO_2_ are shown in Supplementary Fig. 17. The spectrum shows two distinguished peaks corresponding to the Cu-O and Cu-Pd. To confirm the characteristics of Cu-Pd rather than Cu-Cu, the EXAFS of Cu K-edge for Cu foil was also fitted (Supplementary Fig. 18). The fitting parameters, e.g., R-bond length, CN-coordination numbers are listed in Supplementary Table 4. The bond length of Cu-Pd is determined to be 2.76 Å with a coordination number of 1.5, larger than that of Cu-Cu bond (2.54 Å) in Cu foil. Such difference is similar to previous reports^39,40^, which proves the successful formation of PdCu alloy. After the reaction, PdCu/TiO_2_ exhibits a slightly positive shift compared with pristine PdCu/TiO_2_ (Fig. 2g), suggesting a higher oxidation degree^38^. The EXAFS spectrum was further fitted to provide more information (Supplementary Fig. 19 and Supplementary Table 4). Similar to the original PdCu/TiO_2_, PdCu/TiO_2_ after the photocatalytic reaction shows distinguished peaks corresponding to the Cu-O and Cu-Pd. However, the coordination number of Cu-O increases after the reaction, corresponding to further oxidation during the reaction system. It is reasonable to see a higher oxidation degree in our system since PdCu is proved as a photohole acceptor later, which can be oxidised readily.

### Charge transfer pathway

**Fig. 3:**
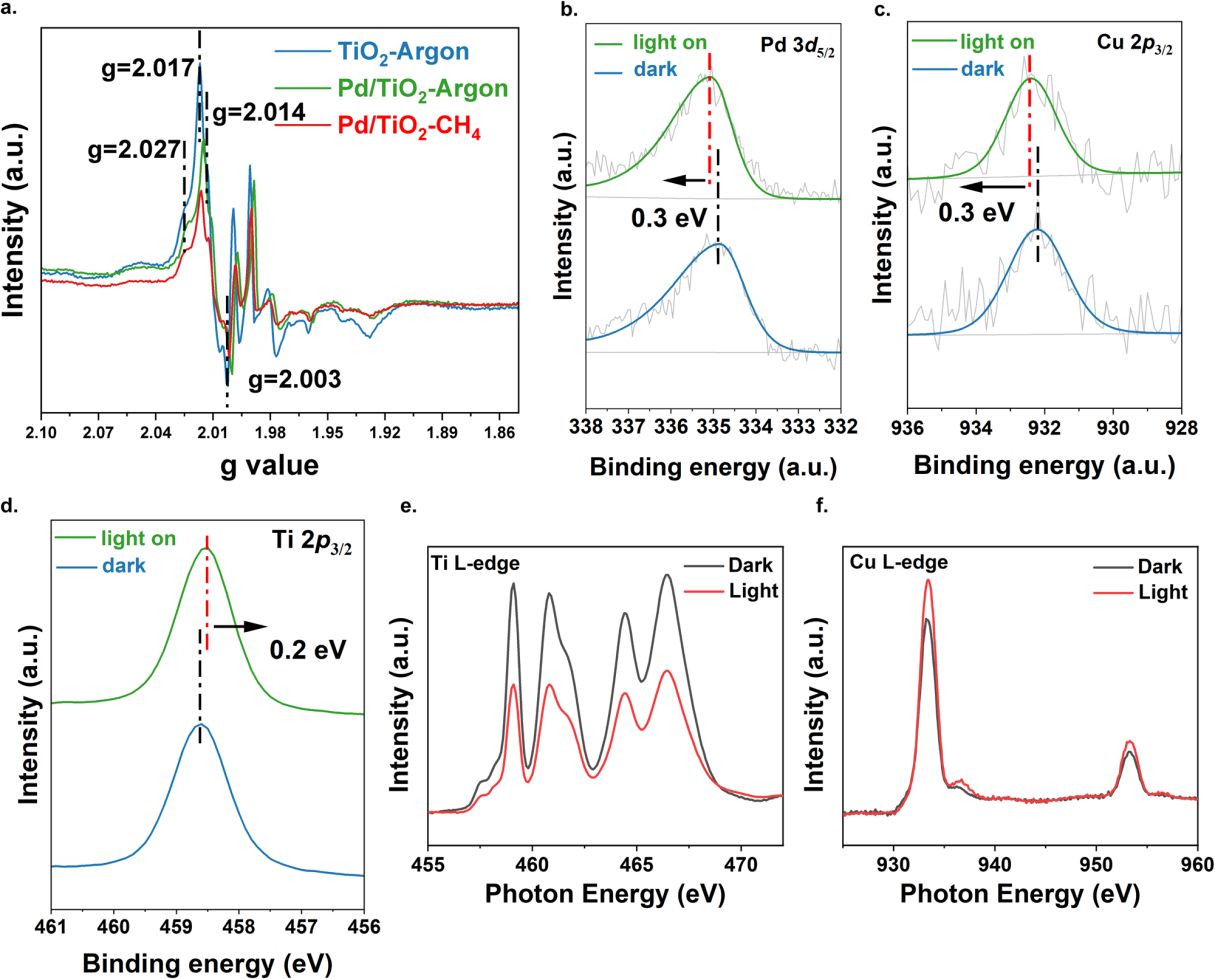
Investigation of photon-induced charge transfer. **a** In-situ EPR spectra of TiO_2_ and Pd/TiO_2_ in argon atmosphere and CH_4_ atmosphere upon light irradiation. **b** In-situ Pd 3*d*_5/2_ XPS spectra of PdCu/TiO_2_ under the dark condition and under light irradiation. **c** In-situ Cu 2*p*_3/2_ XPS spectra of PdCu/TiO_2_-(1:9) under the dark condition and light irradiation condition. **d** In-situ Ti 2*p*_3/2_ XPS spectra of PdCu/TiO_2_-(1:9) under the dark condition and light irradiation condition. **e** Ti L-edge NEXAFS spectra of PdCu/TiO_2_ under dark condition and light irradiation. **f** Cu L-edge NEXAFS spectra of PdCu/TiO_2_ under dark condition and light irradiation. Source data are provided as a Source Data file.

The charge transfer process of PdCu/TiO_2_ was investigated via in-situ techniques (EPR, XPS, and NEXAFS). Under dark conditions (Supplementary Fig. 20), the EPR spectrum of pristine TiO_2_ itself exhibits some signals related to the small amount of surface oxygen species (e.g., adsorbed superoxide)^41^. Compared with this, two sets of new paramagnetic species appear under light irradiation (Fig. 3a and Supplementary Fig. 20). One set of g values (2.003, 2.014, 2.017) can be attributed to the bridge oxygen radical (e.g., Ti^4+^−O^·-^−Ti^4^) and another set of g values (2.003, 2.017, 2.027) is owing to the terminal oxygen radical (e.g., Ti^4+^−O^2-^−Ti^4+^−O^·-^), which is equivalent to photoholes located on O^2-^ anions^42–44^. After loading of Pd nanoparticles, the intensity of both sets shows an obvious decrease (Fig. 3a), indicating the transfer of photoholes from TiO_2_ to Pd nanoparticles or the photoholes in TiO_2_ being consumed by the electrons from the d-band of Pd. Moreover, when the argon atmosphere was replaced by a methane atmosphere in the in-situ EPR measurement, the intensity of these two sets of g values was reduced further. Since methane can work as a photohole scavenger to consume photoholes, this result implies that the Pd shows a similar function to methane, which is the photohole acceptor.

The valence-band XPS was used to identify the valence band maximum (VBM) of TiO_2_ before and after loading the nanoalloy cocatalyst. Supplementary Fig. 21 shows that some metal-induced occupied states (metallic d-orbital structure) are formed on the VBM of TiO_2_ after the introduction of Pd or PdCu nanoalloy, which may provide additional active sites in photocatalysis as reported previously^45^. These states can trap the photoholes from the valence band of TiO_2_. It was not able to compare the PdCu/TiO_2_ here with other samples, due to the inference from the very strong Cu EPR signal (Supplementary Fig. 22)^46^.

In-situ XPS was carried out to further confirm the charge transfer from TiO_2_ to PdCu (Fig. 3b–d). A positive shift of ~0.3 eV for the Pd 3*d*_5/2_ peak is clearly observed during light irradiation (Fig. 3b), showing direct evidence that Pd works as a hole acceptor during the photocatalytic reaction. However, the signal for Cu 2*p* can not be identified due to the low weight content. Thus, the sample with a higher Pd/Cu precursor mole ratio (1:9) was used for analysis, since this sample exhibits a similar activity trend as PdCu/TiO_2_ prepared with a Pd/Cu mole ratio of 1:1 (Supplementary Fig. 3). As shown in Fig. 3c, a positive shift of ~0.3 eV can also be observed for Cu 2*p*_3/2_ peak under light irradiation, suggesting that Cu also works as a hole acceptor during the photocatalytic reaction. In contrast to Cu and Pd, Ti 2*p*_3/2_ peak exhibits a negative shift of 0.2 eV under light irradiation (Fig. 3d). This further supports that photoholes are transferred to PdCu and photoelectrons are left on TiO_2_.

To further verify the role of the Cu species, the in-situ near-edge X-ray absorption fine structure (NEXAFS) spectra were carried out, as shown in Fig. 3e, f. The NEXAFS spectra are sensitive to reflect the density of states of the orbitals and the Ti, Cu L-edge NEXAFS spectra of PdCu/TiO_2_ under dark and light illumination were measured. The Ti L-edge and Cu L-edge NEXAFS arise from the 2*p*−3*d* electron transition. Under light illumination, the electrons will be excited from the valence band to the conduction band in TiO_2_. The conduction band consists of Ti 3*d* orbital, thus the Ti L-edge NEXAFS feature weakens due to the decreasing density of unoccupied Ti 3*d* states after receiving the electrons from the valence band (Fig. 3e)^47^. In contrast, the intensity of Cu L-edge NEXAFS spectra enhances under light irradiation, suggesting an increasing density of unoccupied Cu 3*d* states^48^. In other words, the photoholes can be transferred from the valence band of TiO_2_ to Cu in PdCu, resulting in the increasing density of unoccupied Cu 3*d* states.

The DFT simulations were carried out to provide theoretical support for this role assignment (detailed discussion is shown along with Supplementary Fig. 23 and Supplementary Table 5).

Based on the above discussion, it is reasonable to propose the PdCu nanoalloy as the photohole acceptor. This seems to be beyond the conventional concept in photocatalysis that the metallic nanoparticle on a semiconductor works as an electron acceptor at first glance. However, it should be noted that the conventional concept was proposed based on the work function for the bulk metals. The real work function for small nanoparticles is hard to determine so far due to the limited resolution (~µm) of the probe/beam^49,50^. As the size of PdCu in our photocatalyst is ~2.3 nm, which is far from this range, the direct application of the band-alignment theory using the value of bulk metal might be inappropriate. The band-alignment between nanoparticles and semiconductors can be affected by the preparation methods (e.g., photodeposition, wet impregnation, precipitation and colloidal synthesis) and the Schottky barrier is not always present^51^. Thus, different roles (hole/electron acceptor) of same metallic nanoparticle prepared by different techniques are often observed. An advanced four-dimensional STEM technique was developed recently to visualise the charge distribution in metallic Au nanoparticles on SrTiO_3_, indicating that even the post-synthesis treatments of the same nanoparticle could vary the charge transfer behaviour (positive charges accumulation or negative charges accumulation) during the reaction^52^. Moreover, the accumulation of charges (photoelectrons/photoholes) on the metallic nanoparticle was found to change according to its specific local environment via the direct observation of Kelvin probe force microscopy under illumination^50^. These recent results indicate that particular attention should be paid when proposing the charge transfer pathway^51^. Actually, metal species as hole acceptors have also been proved recently via in-situ XPS, FT-IR, and operando DRIFT, e.g., Pd/TiO_2_ in nonoxidative coupling of methane^53^, Pt/Ga_2_O_3_ and Pd/Ga_2_O_3_ in photocatalytic oxidation of methane with water^54^.

The enhanced charge separation was further proved by photoluminescence (PL) and transient photocurrent response (Supplementary Figs. 24 and 25 with discussion alongside). As the photocatalytic OCM reaction involves two redox reactions (methane oxidation by photoholes and oxygen reduction by photoelectrons), the electrochemical oxygen reduction experiments were carried out (Supplementary Fig. 26). All samples present a similar onset potential, because the co-catalysts work as the hole acceptors in all cases and the active sites for oxygen reduction should be located on TiO_2_ itself. The enhanced reduction current density after loading of co-catalysts can be attributed to the efficient charge transfer.

The isotope labelling experiment using ^18^O_2_ as feedstock gas was carried out to double-confirm this half-reaction. As shown in Supplementary Fig. 27a, b, the H_2_^16^O peak (m/z = 18) intensity shows a much higher intensity compared with that in the background mass spectra. This result suggests the formation of water during the OCM experiment. To distinguish the oxygen source from TiO_2_ or O_2_, the isotope labeling experiment using ^18^O_2_ as the feedstock gas was carried out (Supplementary Fig. 27c). Expectedly, the m/z of 18 assigned to H_2_^16^O shows similar intensity in both background mass spectra and the mass spectra using ^18^O_2_ as the feedstock. Furthermore, the new signal (m/z = 20) corresponding to H_2_^18^O appears using ^18^O_2_ as the feedstock, indicating the formation of water from the O_2_ reduction during the reaction.

### Reaction mechanism

After the investigation of the charge transfer, DFT calculations were carried out to investigate the interface reactions. Methane molecules show a final adsorption structure with three C-H bonds pointing down to the surface and one C-H bond pointing up (Supplementary Fig. 28). The length of the C-H bond in the highly symmetric methane molecule in the gas phase was calculated as 1.099 Å and there is almost no change of the C-H distance after adsorption on TiO_2_ (Supplementary Table 6). In contrast, the length of C-H (pointing down) could be up to 1.102–1.106 Å when methane was on the surface of Pd or PdCu, indicating a stronger interaction between H of methane and Pd or PdCu than that between H of methane and TiO_2_. Such softened C-H bonds in methane through the interaction with the Pd surface have also been observed previously^25^, and they were easier to be activated, in particular beneficial for the holes with a lower oxidative potential in Pd.

**Fig. 4:**
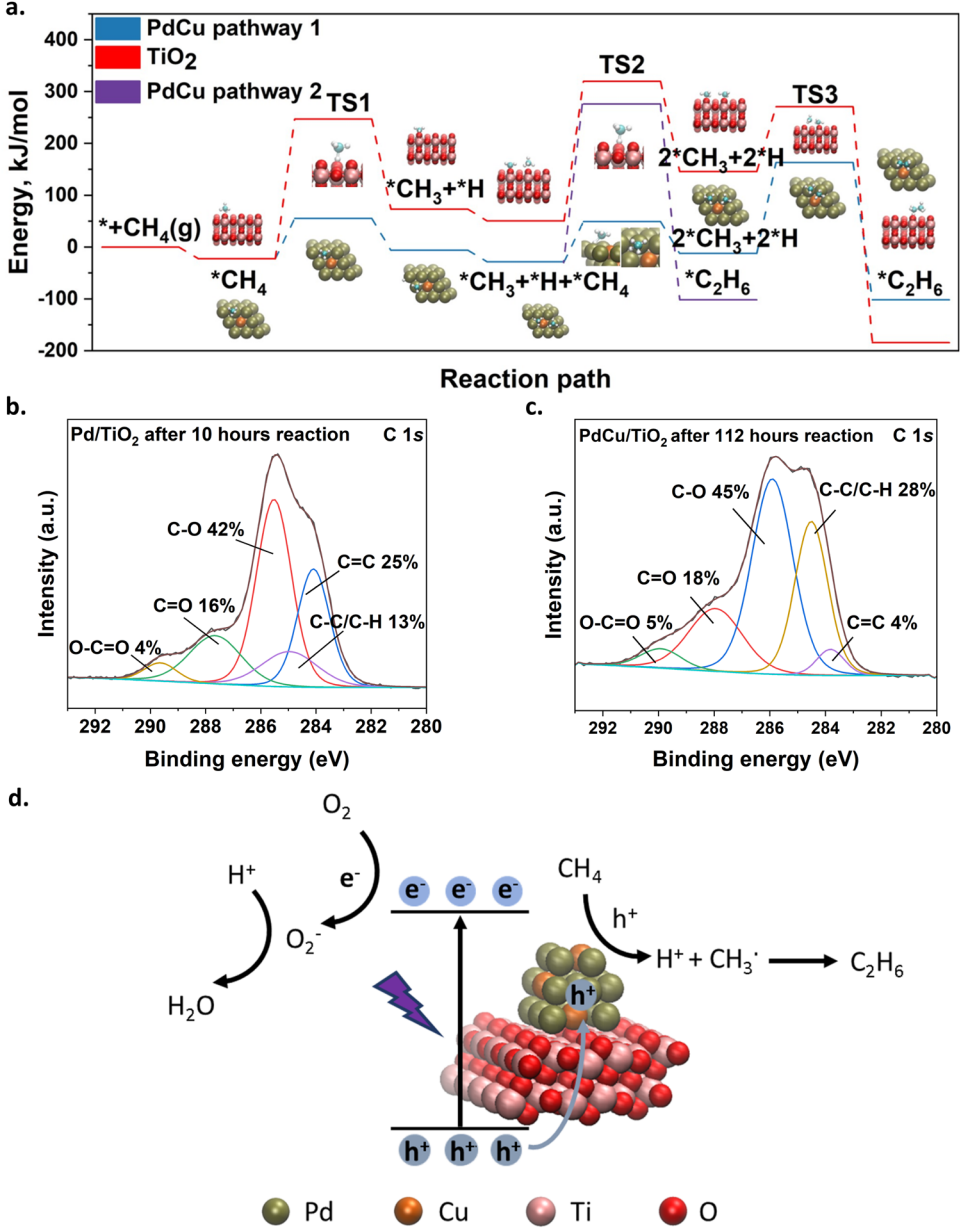
Investigation of photocatalytic mechanism. **a** Calculated energy diagrams for methane conversion to ethane on PdCu and TiO_2_. Apart from the methyl radicals coupling pathway (blue line and red line, pathway 1), another pathway (pathway 2, purple line) via the coupling of methyl radicals and methane molecule to form ethane on PdCu is also presented. The ball models for the main pathway on both PdCu and TiO_2_ are shown (red – O, pink – Ti, tan – Pd, orange – Cu, teal – C, white – H) **b** XPS spectra (C 1 *s*) of Pd/TiO_2_ after 10-hour reaction. **c** XPS spectra (C 1 *s*) of PdCu/TiO_2_ after 112-hour reaction. **d** Scheme of photocatalytic OCM over PdCu/TiO_2_. Source data are provided as a Source Data file.

Then, the reaction barriers for the methane conversion to ethane were determined (Fig. 4a, Supplementary Table 7, 8). The C-H bond in methane requires a large energy (~470 kJ/mol) to cleave it in the gas phase, consistent with previous reports^3^. In contrast, the dissociation of C-H on Pd or PdCu near Pd site has the lowest reaction barrier (TS1) (about 77 kJ/mol), which is more than three times lower than that on TiO_2_ (Supplementary Table 7). Thus, the traditional high reaction barrier of the rate-determining step, C-H activation, can be greatly relieved after the introduction of PdCu. After the cleavage of the C-H bond, the following step can be the abstraction of H of another adsorbed methane molecule (pathway 1) or the direct coupling of the methyl radicals with another adsorbed methane molecule (pathway 2). These two pathways were investigated on different surfaces (Supplementary Table 8). The coupling of two methyl radicals shows the lowest reaction barrier on all surfaces compared with the other two pathways (direct coupling of two methane molecules or coupling one methane with one methyl radical). This pathway is also widely accepted in photocatalytic methane conversion to ethane^2^. In particular, the activation barrier for TS2 of pathway 2 is 130 kJ/mol larger than that of pathway 1 on PdCu, as shown in Fig. 4a. Therefore, the formation of ethane is supposed to follow the coupling of two methyl radicals formed from two separately adsorbed methane molecules. The activation barrier for methyl radicals coupling (TS3) on three catalysts is similar (Supplementary Table 8). This barrier is much less than the C-H activation barrier on TiO_2_ (269.5 kJ/mol), indicating that the methane conversion to ethane process on TiO_2_ is greatly limited by the rate-determining step of C-H activation and bond dissociation of  two separate methane molecules. Therefore, the introduction of Pd can promote the activation of CH_4_ and this effect can be maintained after the formation of PdCu nanoalloy.

However, the strong interaction between the reactant and the active sites may also result in the stronger binding of as-formed products (e.g., C_2_H_6_ in this work), mitigating their desorption and accelerating coking that would block the active sites to cause the catalyst deactivation. This is likely to explain the declining activity trend of Pd/TiO_2_ (Supplementary Fig. 5). To examine this assumption, the Raman spectroscopy using a higher energy laser (enhanced by a factor of ten) was carried out to detect coke species in the range of 1000–3000 cm^−1^ (Supplementary Fig. 29). Before the reaction, the Pd/TiO_2_ exhibits two peaks with low intensity at around 1350 (D mode) and 1600 cm^−1^ (G mode) and a very small peak at ~2900 cm^−1^ related to the combination of two modes under very strong laser excitation, respectively, which could be attributed to the tiny residue of amorphous carbon adsorbed on the TiO_2_ photocatalyst^55–58^. After only 5 h of reaction over Pd/TiO_2_, all peak intensities are strongly enhanced. These typical bands confirm the formation of cokes during photocatalytic OCM reaction over Pd/TiO_2_, resulting in rapid deactivation. In contrast, such enhancement of peak intensities cannot be observed over the optimised catalyst PdCu/TiO_2_ even after the 112-hour stability test, suggesting that the introduction of Cu can enhance coke resilience.

The XPS (C 1 *s*) has also been conducted to investigate the coke formation of Pd/TiO_2_ and PdCu/TiO_2_ after reactions (Fig. 4b, c). The high-resolution C 1 *s* spectra can be deconvoluted into five C species, such as C = C (ca. 284.3 eV), C-H/C-C (ca. 284.8 eV), C-O (ca. 285.6 eV), C = O (ca. 288.0 eV) and O-C = O (ca. 290.0 eV)^59,60^, and their proportions are also provided for comparison. Both used catalysts show similar proportions of C-O, C = O, and O-C = O. The C-O can be attributed to the adsorption of some methyl radicals on the surface of TiO_2_ and the C = O or O-C = O may be related to the overoxidation of methane to CO_2_. Strikingly, these two spent catalysts exhibit differences in the content of C-C/C-H (sp^3^) and C = C (sp^2^). The ratio between sp^2^ C /sp^3^ C is ca. 2 and the content of sp^2^ C is as high as 25% over Pd/TiO_2_ after only 10 hours of reaction, while the ratio decreases to 0.14 with only 4% of sp^2^ C can be observed over PdCu/TiO_2_ even after 112-hour reaction. This indicates that the introduction of Pd can promote dehydrogenation and the C = C species is likely attributed to the formation of cokes. In contrast, Cu may weaken the interaction of the co-catalyst and as-formed products, avoiding the consecutive dehydrogenation to form cokes.

It should be noted that adventitious carbon species can somewhat affect the XPS C 1 *s* spectrum^61^. Thus, the TGA-MS was also carried out to have an unambiguous confirmation of coking. The solid coke species on Pd/TiO_2_ likely cover the active sites to deactivate Pd/TiO_2_ due to the deep dehydrogenation of CH_4_ to C species, while the introduction of Cu can avoid the consecutive reaction to form coke (detailed discussion alongside Supplementary Fig. 30). This is consistent with the Raman and XPS results. Similar strategies to use Cu to form an alloy to enhance coke resistance via weaken binding to adsorbates (e.g., ethane in our cases) have been reported in thermocatalysis^17,21,22,62^.

DFT calculations were conducted to gain an in-depth understanding of ethane adsorption on Pd and PdCu (the optimised process of different adsorption structures is shown in Supplementary Figs. 31 and 32). As expected, the direct involvement of Cu atoms effectively decreases the adsorption energies of the ethane molecule (Supplementary Table 9), by up to ca. 4 kJ/mol, suggesting the as-formed ethane products can be more easily released from PdCu than from pure Pd, avoiding the consecutive reactions to form cokes. A similar strategy was recently used with the isolation of the Pd site by other components to inhibit coke accumulation in a hydrogenation reaction^37^. Thus, it is reasonable to witness an unchanged activity after even 112 h of reaction over PdCu/TiO_2_ using the nanoalloy. DFT simulations were also carried out to investigate the catalyst deactivation from the loss of lattice oxygen in TiO_2_ and the result suggests that this is not the case in our aerobic reaction system (detailed discussion was shown alongside Supplementary Table 10–12).

A reaction mechanism for photocatalytic OCM reaction over PdCu/TiO_2_ was proposed as shown in Fig. 4d. Upon light irradiation, photoelectrons excited from the valence band (VB) of TiO_2_ to the conduction band (CB) would reduce oxygen to form superoxide radicals on TiO_2_, while photoholes from the valence band of TiO_2_ would transfer to PdCu nanoalloy where methane is adsorbed. The generated positively charged states would break the pre-softened C-H bonds in the adsorbed methane molecules to form methyl radicals and protons. The coupling of methyl radicals produces ethane molecules and some of them may be further dehydrogenated to form ethylene. The protons would then be consumed by the superoxide radicals to generate water, thus completing the cycle. Moreover, the efficient charge transfer and activation of methane after the introduction of Pd nanoparticles would generate more methyl radicals, which also enhances C_2_ selectivity rather than overoxidation to CO_2_ owing to the second-order nature of the coupling reaction to form ethane^63^. The formation of nanoalloy with Cu is important to reduce the adsorption energy of the product, thus maintaining a high selectivity to C_2_ and avoiding coking. The synergistic effect of PdCu nanoalloy demonstrates a stable, efficient, and selective photocatalytic OCM cycle.

## Discussion

In summary, we have reported PdCu nanoalloy (~2.3 nm) decorated TiO_2_ as an efficient and stable photocatalyst for OCM in a flow reactor under ambient conditions. The C_2_ yield rate of 62 μmol h^−1^ has been achieved with a space velocity of 342,000 mL g_cat_^−1^ hour^−1^ with a high AQE of ~8.4% at 365 nm thanks to the synergy of Pd and Cu in the nanoalloy. The high stability of the catalyst has also been demonstrated as there is no noticeable decay of the activity during the 112 h test. Thus, both high TON_PdCu_ of 12,642 and TOF_PdCu_ of 116 h^−1^ have been achieved, being a new benchmark in the photocatalytic methane coupling reaction. Fundamentally, the photon-induced holes on TiO_2_ can be effectively consumed by the electrons from PdCu nanoalloy, which not only retard the charge recombination but also generate new oxidative orbitals with lower oxidative potential of the reaction sites to drive selective conversion of methane. In particular, Pd works as the active sites to soften and activate C-H bond in methane, while Cu enhances the coke-resilience of the catalysts by decreasing the adsorption energy of as-formed products. This work shows that the assembly of multifunction nanoalloys can be a promising strategy to meet the challenges of efficiency and stability in photocatalytic methane conversion.

## Methods

### Photocatalysts fabrication

A modified reduction method by NaBH_4_ was used to prepare noble metals (Pd, Ru, Pt, Ag) decorated TiO_2_^64^. In a typical experiment, different amounts of PdCl_2_ (Aldrich, 99.999%) aqueous solution (2.7 mg_Pd_/mL) was added to 100 mL of deionised water under stirring. Then, a polymer stabilizer, Poly (vinyl alcohol) (PVA, Aldrich, Mw 9000–10000, 80% hydrolyzed) aqueous solution (1%wt) was added with a weight ratio (polymer/metal) of 0.65. Afterward, certain amount of fresh NaBH_4_ (Aldrich, ≥96%) aqueous solution (0.1 M) was added with a mole ratio (NaBH_4_/Pd) of 5 and the solution was stirred for another 30 min. Next, the concentrated H_2_SO_4_ (0.25 mL) was introduced to the above solution under stirring along with 250 mg of TiO_2_ (Millennium PC-50). After stirring for another 1 h, the photocatalyst was obtained by centrifugation, washed with deionsied water for three times, and dried in the oven for 12 h at 60 °C. The photocatalyst synthesized by this method was denoted Pd_x_/TiO_2_ (x = 0.05, 0.1, 0.5, 1.0, wt%, corresponding to the added amount of Pd weight percentage with respect to TiO_2_). The other noble metals decorated TiO_2_, denoted M/TiO_2_ (M = Pt, Ag, Ru, Au), were synthesized using the similar method with an equal molar amount to Pd in Pd_0.1_/ TiO_2_.

For the synthesis of bimetallic alloy decorated TiO_2_ photocatalyst, denoted PdM/TiO_2_ (M= Cu, Ni, Co, Fe), a modified method was used^65^. Typically, 0.25 mg Pd in the form of PdCl_2_ aqueous solution (2.7 mg_Pd_/mL) was added to 45 mL of deionised water, followed by the addition of the equal molar amount of Cu(NO_3_)_2_•2.5H_2_O (Alfa Aesar, 98%) aqueous solution. Subsequently, PVA aqueous solution (1%wt) was added with a weight ratio (polymer/Pd) of 1.3, which is two times the amount added in the synthesis of Pd_0.1_/TiO_2_. In addition, a certain amount of sodium citrate (Sodium citrate tribasic dihydrate, Sigma-Aldrich, ≥99.0%) aqueous solution (1.4 mg/mL) was mixed at a molar ratio of sodium citrate to metal at 2: 3. Then, the above mixture was purged with argon for more than 20 min under stirring. After the removal of air, a certain amount of fresh NaBH_4_ aqueous solution (0.1 M) was injected into the above solution with a molar ratio of NaBH_4_/Pd to10 and the solution was stirred for another 1 h at room temperature with continues argon flow. Afterward, 250 mg of TiO_2_ was added to the above solution and the mixture was allowed to stir for another 0.5 h. The photocatalysts were collected by centrifugation, washed three times with deionised water, and dried in a vacuum oven overnight. For the other metals (Ni, Co, Fe), a similar procedure was conducted except for the change of the metal nitrate precursors. For the synthesis of controlling samples Cu/TiO_2_, a similar procedure was conducted apart from the introduction of related metal precursors. For the synthesis of the sample (Pd_1_Cu_1_/TiO_2_-large) with a large loading amount (1%wt of Pd and equal molar amount of Cu), a similar procedure was carried out except the ten times higher amount of related chemicals.

### Characterisation

The powder X-ray (XRD) spectra were measured with a Rigaku SmartLab SE using a Cu Kα1 source (60 kV, 60 mA). Raman spectroscopy was performed at Renishaw InVia Raman Microscope with a 442 nm laser. Ultraviolet-visible diffuse reflectance spectroscopy (UV-Vis DRS) was recorded by a SHIMADZU UV-2550 in reflectance mode using standard BaSO_4_ powder as a reference. UV-Probe software was used to convert the reflectance to absorbance via Kubelka-Munk transformation. XPS Analysis was carried out in two instruments. Most samples were measured using Kratos Axis SUPRA XPS while the Cu 2*p* of PdCu/TiO_2_-large were measured with a Thermoscientific XPS K-alpha with more scans to obtain high signal-to-noise spectra. In-situ XPS experiment was conducted using a Thermo Fisher ESCALAB 250Xi equipped with a 365 LED light source. All the XPS analysis was performed with CasaXPS software and the spectra were calibrated with C 1 s peak at 284.8 eV. PL spectroscopy was collected by Renishaw InVia Raman with a 325 nm excitation laser. The X-ray absorption spectra (XAS) were collected at the 4B9A beamline in Beijing Synchrotron Radiation Facility (BSRF), China. The storage rings of BSRF were carried out at 2.5 GeV and a stable current of 400 mA. The data collection was conducted in fluorescence mode under ambient conditions using a Si (111) double-crystal monochromator and a Lytle detector. Data analysis and fitting were performed by the Athena and Artemis programs of Demeter software packages using the FEFF6 program^66,67^. A standard Cu foil was used for the energy calibration which was measured as a reference. A linear function was subtracted from the pre-edge region, then the edge jump was normalised using Athena. For EXAFS modelling, the global amplitude EXAFS (e.g., coordination numbers, the distance to the neighboring atom) were analyzed by nonlinear fitting of the Fourier-transformed data in *R*-space using Artemis. The EXAFS of the Cu foil was first fitted and the obtained amplitude reduction factor *S*_*0*_^*2*^ value was applied to analyse the coordination numbers in the Cu-O/Pd scattering path in PdCu/TiO_2_. STEM imaging was conducted using a double Cs aberration-corrected FEI Titan^3^ Themis 60-300 equipped with an X-FEG gun, a monochromator, and an XEDS ChemiSTEM. The element content was measured by a USA Perkin Elmer 8300 ICP-OES and a Z-2000 HITACHI Atomic Absorption Spectrophotometers (AAS). In-situ EPR was measured using an NRUKER E500-9.5/1.2 spectrometer with a 365 nm LED light source (71 W) under argon, air and CH_4_ atmosphere, respectively at 100 K. During the EPR experiments, powder samples (~13 mg) were added in quartz tubes and sealed with septa, then purging by different atmosphere. In-situ Near-edge X-ray absorption fine structure (NEXAFS) spectra were collected at Photoemission End-station (BL10B) in the National Synchrotron Radiation Laboratory (NSRL) in Hefei, China. The data collection was carried out in the total electron yield mode under ambient conditions with a 320 nm LED placed 15 cm in front of the sample. The photon energy was calibrated by the 4 *f* spectral peak of a freshly sputtered gold wafer.

### (Photo)electrochemical measurements

The electrochemical oxygen reduction reactions were conducted on a Metrohm Autolab potentiostat with a three-electrode system. A photocatalyst-loaded glassy carbon rotating disk (RDE, 3 mm diameter) was used as a working electrode, a glassy carbon rod worked as a counter electrode and Hg/HgO (1 M NaOH) was used as a reference electrode in O_2_ saturated 0.1 M of KOH electrolyte, respectively. For the preparation of the working electrode, 4 mg of photocatalysts powder was added into 1 mL of suspension containing 700 μL of deionised water, 264 μL of ethanol, and 36 μL of nafion solution (Sigma-aldrich, Nafion® 117 solution, 5 w/w). After sonication for 30 minutes, a homogenous ink was obtained and 5 μL of the ink was deposited onto the glassy carbon RDE, followed by rotating drying for 1 h under room temperature. Linear sweep voltammograms (LSV) were carried out at a rotation rate of 1600 rpm and a scan rate of 10 mV s^−1^ in the voltage range of 0.2–1.1 V vs. RHE. The photocurrent investigation was carried out on Gamry Instrument Interface 5000E potentiostat under chopped illumination. Typically, 10 μL of the ink prepared using the same recipe as that used in the oxygen reduction reaction was spin-coated on a 0.5 × 1 cm FTO glass with an exposure area of 0.5 × 0.5 cm at 500 rpm for 0.5 min followed by 1000 rpm for 4 min. The process was repeated 3 times. After drying at 80 °C for 120 min, the photocatalyst coated FTO electrode was installed as a working electrode, the Pt plate worked as the counter electrode and Ag/AgCl/3 M KCl was used as the reference electrode in 0.1 M Na_2_SO_4_ electrolyte. The applied potential was set as 0.2 V vs. Ag/AgCl and a 450 W Xe lamp equipped with an AM 1.5 G filter was used as the light source to maintain the light intensity at 100 mW cm^−2^. Samples with a geometric area of around 0.25 cm^2^ were illuminated from the front side (electrode-electrolyte side). The light illumination was chopped for every 20 s during the whole measuring period.

### Photocatalytic reaction for methane conversion

The photocatalytic OCM reaction was conducted in a flow reactor (Supplementary Fig. 33) equipped with a 365 nm LED (Beijing Perfect Light, PLS-LED 100). In a typical experiment, 50 mg of sample was dispersed in 50 mL of deionized water and the mixture was allowed to sonicate for at least 15 min until to achieve a good dispersion. Afterward, the mixture was filtered with a membrane (glass fibre, diameter 37 mm) to obtain a uniform film. After drying the film in the oven, the film was added in the reactor and fixed with a stainless ring, followed by the sealing with a rubber O-ring and a cover. The thermocouple was inserted from the bottom of the reactor and its probe could reach the position of the catalyst bed. Then, the temperature of the system was monitored via the control panel during the reaction. The temperature was 30 °C during the reaction. Three mass flow controllers (MFC, Bronkhorst) were used to control the flow rates of air (BOC, 99.999%), methane (10%, v/v, methane/argon, BOC, 99.999%), and argon (BOC, 99.999%), respectively. To investigate the influences of different ratios of CH_4_ to O_2_, the real ratio was tuned to 53:1, 114:1, 176:1, and 373:1, respectively after a few calibrations (excluding the influence of temperature and gas line pressure). Before each photocatalytic OCM reaction, the system was purged using the gas mixture at a certain ratio to reach an equilibrium (approximately 90 min). Then, the 365 nm LED (40 W) irradiated from the top quartz window of the reactor and the outlet gases were flowed to two different GCs (Agilent 7820 and Varian 450) to online quantify all the gas products. The permanent gases (e.g., N_2_, O_2_, H_2_, CH_4_) were firstly separated from other heavier gas components (e.g., CO_2_, C_2+_ products) by a CarbonPlot column, then separated on a Molesieve 5 A column for a final analysis by the thermal conductivity detector (TCD) of Agilent 7820. The organic products, e.g., C_2_H_4_, C_2_H_6_, and C_3_H_8_ was analysed by the flame ionization detector (FID) with an HP-PLOT Q column in the Agilent 7820 machine. The FID in the Varian 450 GC was equipped with a methaniser to quantify the low concentration of CO_2_ and CO (*Note*: some work used a TCD detector to analyse CO and CO_2_ instead of an FID detector with a mechaniser, the former has a much lower sensitivity than the latter and might lead to an overestimation of the selectivity). For the long-term experiment running, the ratio of CH_4_ to O_2_ was set as 373: 1, and the experiment was started after reaching equilibrium in each morning and stopped in the evening. (*Note*: Due to the safety requirement, the overnight run was not allowed with such high CH_4_/O_2_ flow rates. Therefore, the long-time run was carried out by consecutive 20 days run with each day on average 6-hour reaction time).

### Calculation of the C_2_ selectivity

The selectivity was calculated based on the observable products, including C_2_H_6_, C_2_H_4_, C_3_H_8,_ and CO_2_. The C_2_ selectivity was calculated using the formula below:

$${C}_{2}\,{selectivity}=\frac{2\times ({n}_{C2H6}+{n}_{C2H4})}{2\times {n}_{C2H6}+2\times {n}_{C2H4}+3\times {n}_{C3H8}+{n}_{{CO}2}} \times 100\%$$

in which n is the molar rate of different products.

### Calculation of apparent quantum efficiency (AQE)

The calculation of apparent quantum efficiency was based on the conversion of methane using the formula shown below:

$${{{{{\rm{A}}}}}}{{{{{\rm{QE}}}}}}=\frac{(2\times {n}_{C2H6}+4\times {n}_{C2H4}+4\times {n}_{C3H8}+8\times {n}_{{CO}2})\times {N}_{A}}{{Number}\,{of}\,{incdient}\,{photons}} \times 100\%,$$

in which n is the molar generation rate of different products, N_A_ is the Avogadro constant, and the irradiation area was approximately 7 cm^2^. The light intensity was 160 mw/cm^2^.

### Calculation of Turnover frequency (TOF) and Turnover number (TON)

The calculation of TON was based on the C_2_ product yields over the active sites PdCu alloy nanoparticle,

$${{{{{\rm{Moles}}}}}}\,{{{{{\rm{of}}}}}}\,{{{{{{\rm{C}}}}}}}_{2}\,{{{{{\rm{yield}}}}}}=5158\,{{{{{\rm{\mu }}}}}}{{{{{\rm{mol}}}}}}$$

$${{{{{\rm{Catalyst\; amount}}}}}}=50\,{{{{{\rm{mg}}}}}}$$

$${{{{{\rm{P}}}}}}{{{{{\rm{dCu}}}}}}\,{{{{{\rm{molar}}}}}}\; {{{{{\rm{amount}}}}}}=\frac{{Catalyst}\,{amount}\times {Pd}\,{concentration}}{{The}\,{atomic}\,{weight}\,{of}\,{Pd}} \\+\frac{{Catalyst}\,{amount}\times {Cu}\,{concentration}}{{The}\,{atomic}\,{weight}\,{of}\,{Cu}}=0.816{{{{{\rm{\mu mol}}}}}}$$

$${{{{{\rm{T}}}}}}{{{{{\rm{ON}}}}}}=\frac{{Moles}\,{of}\,{C}_{2}\,{yield}\times 2}{{PdCu}\,{molar}\,{amount}}={{{{\mathrm{12,642}}}}}$$

$${{{{{\rm{T}}}}}}{{{{{\rm{OF}}}}}}=\frac{{C}_{2}\,{yield}\,{rate}\times 2}{{PdCu}\,{molar}\,{amount}}=116{{{{{{\rm{h}}}}}}}^{-1}$$

Noted: For the TON and TOF of other works presented in Fig. 1e, the calculation was based on the active site, such as noble metal co-catalyst in their stability test periods. For those with more than one co-catalyst, the calculation was based on the sum of the co-catalysts amount used for CH_4_ activation.

### Isotope labelling experiment

For the isotope labelling experiment, a similar photocatalytic process was conducted except ^13^CH_4_ (^13^C enrichment >99% atom, Wuhan Newradar Gas Co.) or ^18^O_2_ (^18^O enrichment >99% atom, Wuhan Newradar Gas Co.) was used as the feed gas. Typically, 20 mg PdCu/TiO_2_ photocatalyst has been used in a flow reactor and a total flow rate of 120 mL/min^−1^ (CH_4_: O_2_ = 80: 1) was introduced under 300 W Xe lamp irradiation. The products containing C-isotope/O-isotope was analysed by an on-line MS (QIC-20, Heiden Analytical Ltd.).

### Computational details

Density-functional theory (DFT) calculations were performed using CRYSTAL17 software^68^, with the PBE functional^69^ and the D3 dispersion correction^70^, with a localised basis set and pseudopotential for Pd^71^, and localised all-electron basis sets for Cu^72^, Ti, O^73^, C and H^74^ obtained from the CRYSTAL web site^75^. Pd and PdCu alloy were modeled using 2D-periodic (111)-oriented stabs with three atomic layers and a 3 × 3 extended surface unit cell, using the lattice parameters optimised for bulk Pd, with slabs separated by a 500 Å vacuum gap. In the PdCu alloy, one out of nine Pd atoms in each layer was replaced by a Cu atom. TiO_2_ was modelled as a periodic slab of anatase with the (101) surface orientation and a 2 × 2 extended surface unit cell, with the thickness of 8 atomic layers, using the lattice parameters optimised for bulk anatase, with slabs separated by a 500 Å vacuum gap. Single methane or ethane molecules were adsorbed on one side of each slab, in a range of positions. Optimisation calculations of adsorbates on Pd and PdCu were carried out using a 4 × 4 *k*-point grid sampled using the Monkhorst-Pack method, while optimisations on TiO_2_ were carried out using a 2 × 2 *k*-point grid. All atom positions were fully optimised. Binding energies were calculated including the basis set superposition error correction^76^. Images of the calculated structures were produced using VMD software^77^. Reaction paths were calculated using the distinguished reaction coordinate method implemented in CRYSTAL17, by defining the reaction coordinate as the C-H distance (for C-H dissociation) or the C-C distance (for methane or methyl reaction to form ethane)^78^. To calculate the densities of electronic states in TiO_2_/Pd and TiO_2_/PdCu combined systems, we optimised interfaces containing (a) a finite Pd_24_Cu_3_ nanoparticle (ratio Pd:Cu = 8:1) adsorbed on the (2 × 2)-extended TiO_2_ anatase (101) slab; (b) a commensurate supercell of (4 × 4)-extended three-layer Pd nanosheet adsorbed on the same (2 × 2)-extended TiO_2_ anatase (101) surface. In the latter case, we considered (i) a pure three-layer Pd nanosheet, (ii) the same Pd nanosheet doped with randomly distributed Cu atoms with the ratio Pd:Cu = 9:1, and (iii) the same Pd nanosheet topped with a single layer of Cu. Optimisations were carried out as described above, using the 4 × 4 k-point grid. The density of electronic states of this combined system was then calculated using a 16 × 16 *k*-point grid.

### Statistics and reproducibility

No statistical method was used to predetermine sample size and no data were excluded from the analyses

### Supplementary information

Supplementary information

### Source data

Source Data

## Data Availability

All data supporting the findings of this study are available within the paper, supplementary information files and the provided source data files. Source data are provided with this paper.
