# Supplementary material for: PdCu nanoalloy decorated photocatalysts for efficient and selective oxidative coupling of methane in flow reactors
Source: Nat Commun. 2023 Oct 10;14:6343. doi: 10.1038/s41467-023-41996-y (PMC10564738; doi:10.1038/s41467-023-41996-y)
Supplement: Supplementary file 1 — Supplementary information [file 41467_2023_41996_MOESM1_ESM.pdf]

Supplementary information

PdCu nanoalloy decorated photocatalysts for efficient and selective oxidative coupling of methane in flow reactors

Authors: Xiyi Li[1], Chao Wang[1], Jianlong Yang[2], Youxun Xu[1], Yi Yang[3], Jiaguo Yu[4], Juan J. Delgado[5,6], Natalia Martsinovich[7], Xiao Sun[8], Xu-Sheng Zheng[9], Weixin Huang[8], Junwang Tang[1,10] *

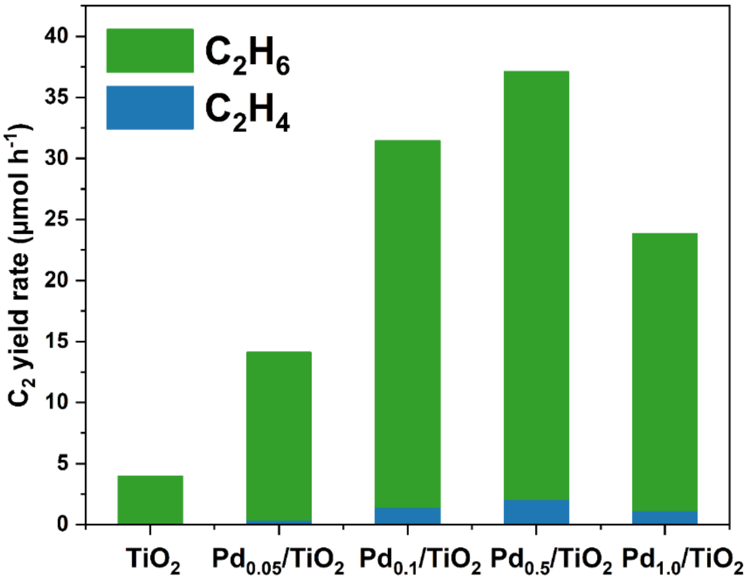

Supplementary Fig. 1 C_2 yield rate of photocatalytic OCM over Pd/TiO_2 with different Pd wt%. (Reaction condition: gas hourly space velocity (GHSV = 342 000 mL g_{cat}^{-1} $hour^{-1}$), CH_4: O_2 = 114: 1, 10% of CH_4, Ar as balance gas, 365 nm LED 40 W, 30 °C)

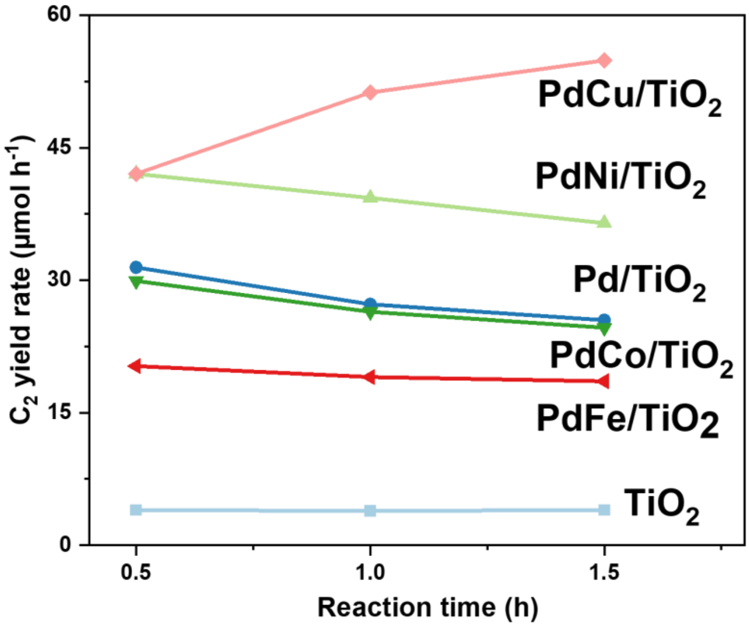

Supplementary Fig.2 The trend of C_2 yield rate over TiO_2, Pd/ TiO_2, PdCu/ TiO_2, PdNi/ TiO_2, PdCo/ TiO_2, and PdFe/ TiO_2 within the first 1.5 hours with the second component of 0.1 wt%. (Reaction condition: gas hourly space velocity (GHSV = 342 000 mL g_{cat}^{-1} hour^{-1}), CH_4: O_2 = 114: 1, 10% of CH_4, Ar as balance gas, 365 nm LED 40 W, 30 °C)

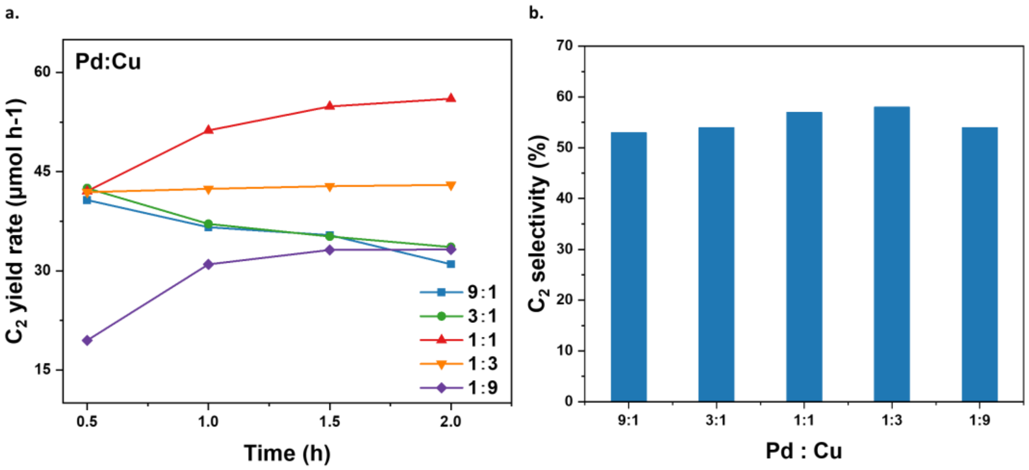

Supplementary Fig.3 a. The trend of C_2 yield rate over 0.1 wt% PdCu modified TiO_2 at different Pd/Cu ratios (9:1, 3:1, 1:1, 1:3 and 1:9) within the first 2.0 hours; b. C_2 selectivity over 0.1 wt% PdCu modified TiO_2 at different Pd/Cu ratios (9:1, 3:1, 1:1, 1:3 and 1:9) for 2.0 hour reaction (Reaction condition: gas hourly space velocity (GHSV = 342000 mL g_{cat}^{-1} hour^{-1}), CH_4: O_2 = 114: 1, 10% of CH_4, Ar as the balance gas, 365 nm LED 40 W, 30 °C)

Interestingly, both Pd/Cu ratios of 9:1 and 3:1 with richer Pd content show a decrease trend of C_2 yield, which is similar to that of Pd/TiO_2 in Supplementary Fig. 2. In contrast, the increasing percentage of Cu (Pd/Cu ratios of 1:1, 1:3 and 1:9) can stabilise the activity. Too much Cu leads to low activity, likely due to the lack of Pd for C-H activation. Thus, the activity at the Pd/Cu optimum ratio (1:1) is the best, together with the nearly highest selectivity. As proved later, the main function of Pd is for C-H activation while the Cu can weaken the adsorption energy of C_2 products to avoid coke accumulation.

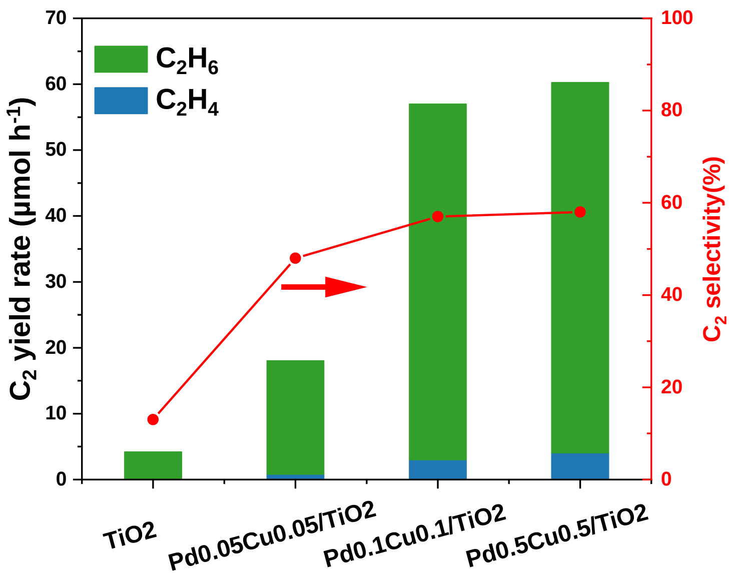

Supplementary Fig.4 The C_2 yield rate and selectivity over PdCu modified TiO_2 with different loading amounts. (Reaction condition: gas hourly space velocity (GHSV = 342 000 mL g_{cat}^{-1} $hour^{-1}$), CH_4: O_2 = 114: 1, 10% of CH_4, Ar as balance gas, 365 nm LED 40 W, 30 °C)

After the determination of the optimum ratio of Pd to Cu (Supplementary Fig. 3), the loading amount effect of PdCu at the ratio of 1:1 was investigated. Compared with pristine TiO_2, the tiny loading of PdCu ($Pd_{0.05}Cu_{0.05}/TiO_2$) shows a great improvement for both the C_2 yield rate (ca. 4 μmol h^{-1} to 18 μmol h^{-1}) and C_2 selectivity (13% to 48%). Further increasing the loading amount of PdCu from 0.05 wt% to 0.1 wt% leads to three times higher C_2 yield rate (from 18 μmol h^{-1} to 57 μmol h^{-1}) with the continuous improvement of selectivity to 57%. However, the slight increase of the yield rate with nearly unchanged C_2 selectivity can be observed with five-fold increase of PdCu loading amount. Balancing the cost and performance of PdCu cocatalyst, 0.1 wt% was then selected as the optimum amount (denoted $PdCu/TiO_2$ unless specifically stated otherwise).

Supplementary Table 1 The weight percentage of Pd and Cu in $Pd_{0.1}Cu_{0.1}/TiO_2$ detected by ICP-OES and AAS.

Element	Pd	Cu
Percentage (wt. %)	0.089	0.051

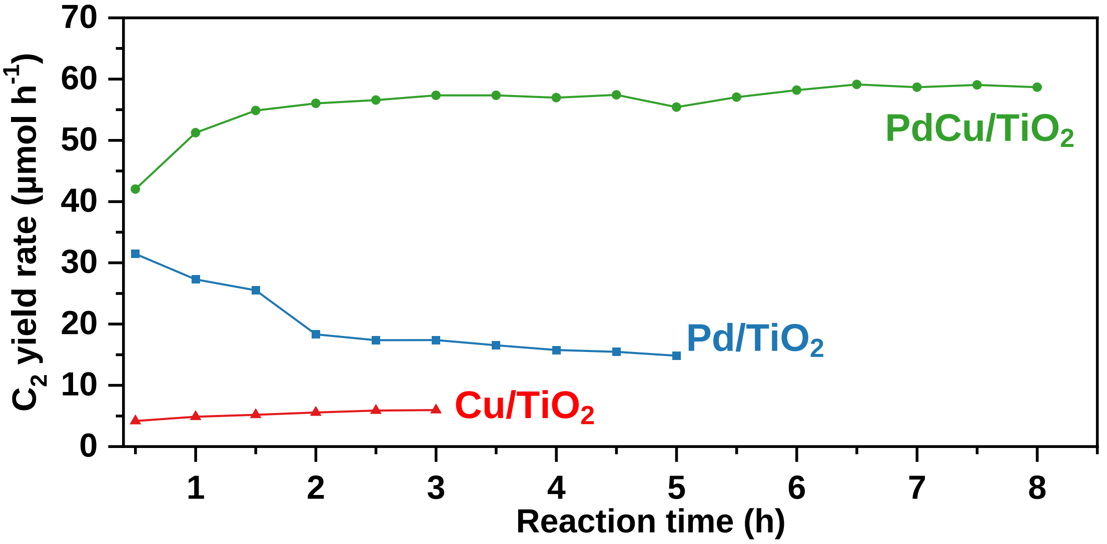

Supplementary Fig.5 The stability and temporal profile of C_2 yield of Pd/TiO_2, Cu/TiO_2, and PdCu/TiO_2. (Reaction condition: GHSV = 342 000 mL g_{cat}^{-1} $hour^{-1}$, CH_4: O_2 = 114: 1, 10% of CH_4, Ar as balance gas, 365 nm LED 40 W, 30 °C)

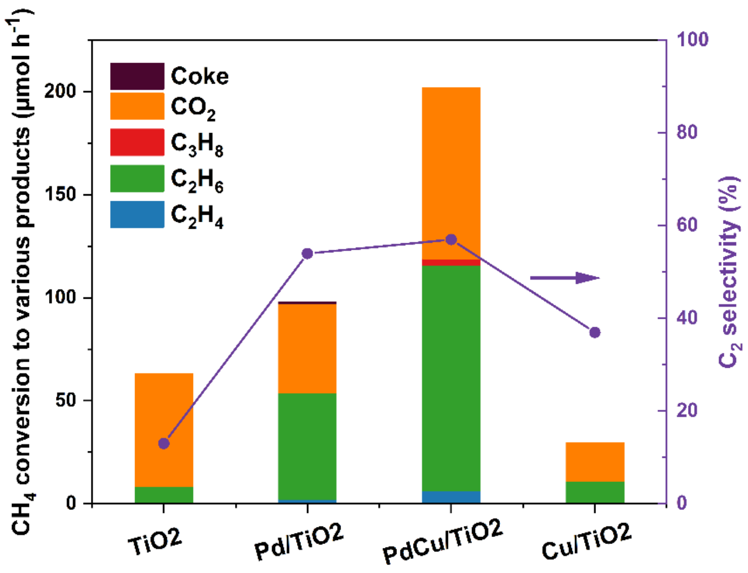

Supplementary Fig.6 CH_4 conversion to various products over TiO_2, Pd/TiO_2, PdCu/TiO_2, and Cu/TiO_2 at 1.5 hour. (Reaction condition: GHSV = 342000 mL g_{cat}^{-1} hour^{-1}, CH_4: O_2 = 114: 1, 10% of CH_4, Ar as balance gas, 365 nm LED 40 W, 30 °C)

Supplementary Table 2 Representative works on photocatalytic methane conversion to ethane/ethylene operated at room temperature.

Samples	Conditions	Yield rate of C_2 products ($\mu mol\ h^{-1}$)	Selectivity of C_2 products (%)	AQE (%)	Ref
$PdCu/TiO_2$	Flow reactor; 303 K; 40 W 365 nm LED; 0.05 g catalyst; $O_2 : CH_4$ = 1 : 53 or 1 : 373, GHSV = 342000 mL g_{cat}^{-1} $hour^{-1}$, 10% of CH_4.	62<br>47.5	52<br>75	8.4 at 365 nm	This work
AuPd/ZnO	Batch reactor; room temperature; 300 W Xe lamp; Reaction time 4 h; 2 mg catalyst; 0.5 mL CH_4.	0.08	99.9	-	[1]
Au/ZnO	Batch reactor; room temperature; 300 W Xe lamp; Reaction time 4 h; 0.5 mL CH_4.	0.01	100	-	[2]
Ga_2O_3-K	Batch reactor; 310 K; 300 W Xe lamp; Reaction time 3 h; 0.2 g catalyst; 200 µmol CH_4	0.054	96	-	[3]
MgO-SiO_2	Batch reactor; 310 K; 300 W Xe lamp; Reaction time 3 h; 0.2 g catalyst; 200 µmol CH_4	0.009	98	-	[4]
Ce-Al_2O_3	Batch reactor; 310 K; 300 W Xe lamp; Reaction time 3 h; 0.2 g catalyst; 200 µmol CH_4	0.1	78	-	[5]
Pd/Ga_2O_3	Flow reactor; 300 K; 40W low-pressure mercury lamp; 0.05 g catalyst; 20 mL min^{-1} CH_4 gas with water vapor	47.4	75.8	5.1 at 254 nm	[6]
Ag-$H_3PW_{12}O_{40}/TiO_2$	Batch reactor; room temperature; 400 W Xe lamp; Reaction time 7 h; 0.1 g catalyst; 0.3 MPa CH_4	2.3	90	3.5 at 365 nm	[7]
Pt-CuO_x/TiO_2	Flow rector; 313 K; 40 W 365 nm LED; 0.1 g catalyst; CH_4: O_2=400: 1, 10%CH_4, GHSV	6.8	60	0.5 at 365 nm	[8]

	= 2400 h^{-1}				
Pt/Ga-TiO_2-SiO_2	Batch rector; room temperature; 300 W Xe lamp; Reaction time 4 h; 0.2 g catalyst; 44.6 µmol CH_4	0.314	90	0.0001 at 350 nm	[9]
Au/TiO_2	Flow reactor; room temperature; 300 W Xe lamp; 5 mg catalyst; 10%CH_4, 90% Ar, GHSV= 120000 mL g^{-1} h^{-1}	0.41	96	-	[10]
TiO_2/SiO_2	Batch rector; 310 K; reaction time 3 h; 300 W Xe lamp; 0.2 g catalyst; 200 µmol CH_4	0.2	90	-	[11]
SiO_2-Al_2O_3-TiO_2	Batch rector; 310 K; reaction time 3 h; 250 W Xe lamp; 1 g catalyst; 200 µmol CH_4	0.69	84	-	[12]
H-MOR	Batch rector; 310 K; reaction time 3 h; 250 W Xe lamp; 1 g catalyst; 200 µmol CH_4	0.074	88		[13]
FSM-16	Batch rector; 310 K; reaction time 3 h; 300 W Xe lamp; 0.2 g catalyst; 200 µmol CH_4	0.018	94		[14]
GaN: ZnO	Batch rector; 293 K; reaction time 2 h; 300 W Xe lamp; 0.05 g catalyst; 300 µmol CH_4	2.85	98		[15]
$Zn_5(OH)_8Cl_2$ H_2O	Batch reactor; 298 K; reaction time 3 h; 300 W Xe lamp; 0.05 g catalyst; pure CH_4	39.4	93		[16]

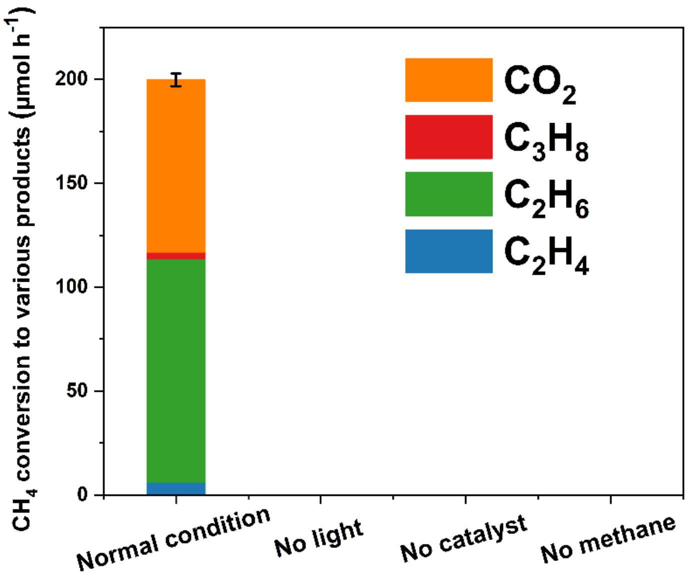

Supplementary Fig.7 Photocatalytic OCM process under various controlling conditions. (Reaction condition: GHSV = 342 000 mL g_{cat}^{-1} hour^{-1}, CH_4: O_2 = 114: 1, 10% of CH_4, Ar as balance gas, 365 nm LED 40 W, 30 °C). Error bar (standard deviation) in the figure was obtained from three sampling runs.

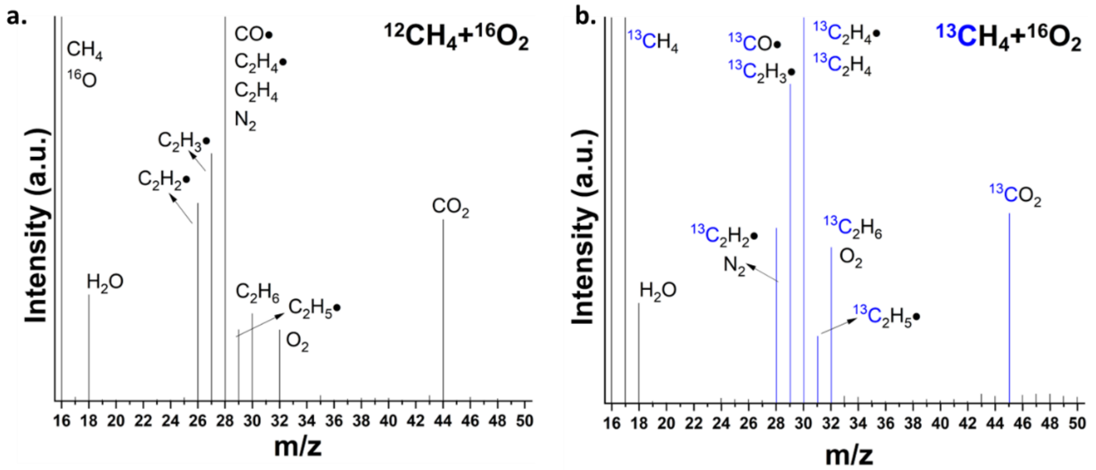

Supplementary Fig.8 Mass spectra of the product using $^{12}CH_4$ (a) or $^{13}CH_4$ (b) as feed gas. (The isotope-labelling products highlighted in blue. The peaks assigned to R· are molecular fragments of the main products. Reaction condition: 20 mg PdCu/TiO$_2$, CH_4: O_2 = 80: 1, total flow rate of 120 mL min^{-1}, 300W Xenon lamp irradiation)

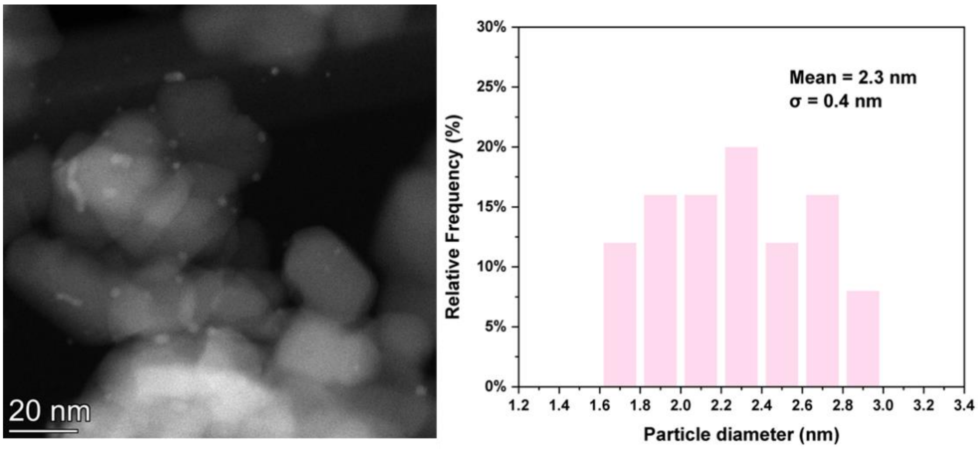

Supplementary Fig. 9 Representative STEM image and particle size distributions of PdCu alloy over PdCu/TiO_2

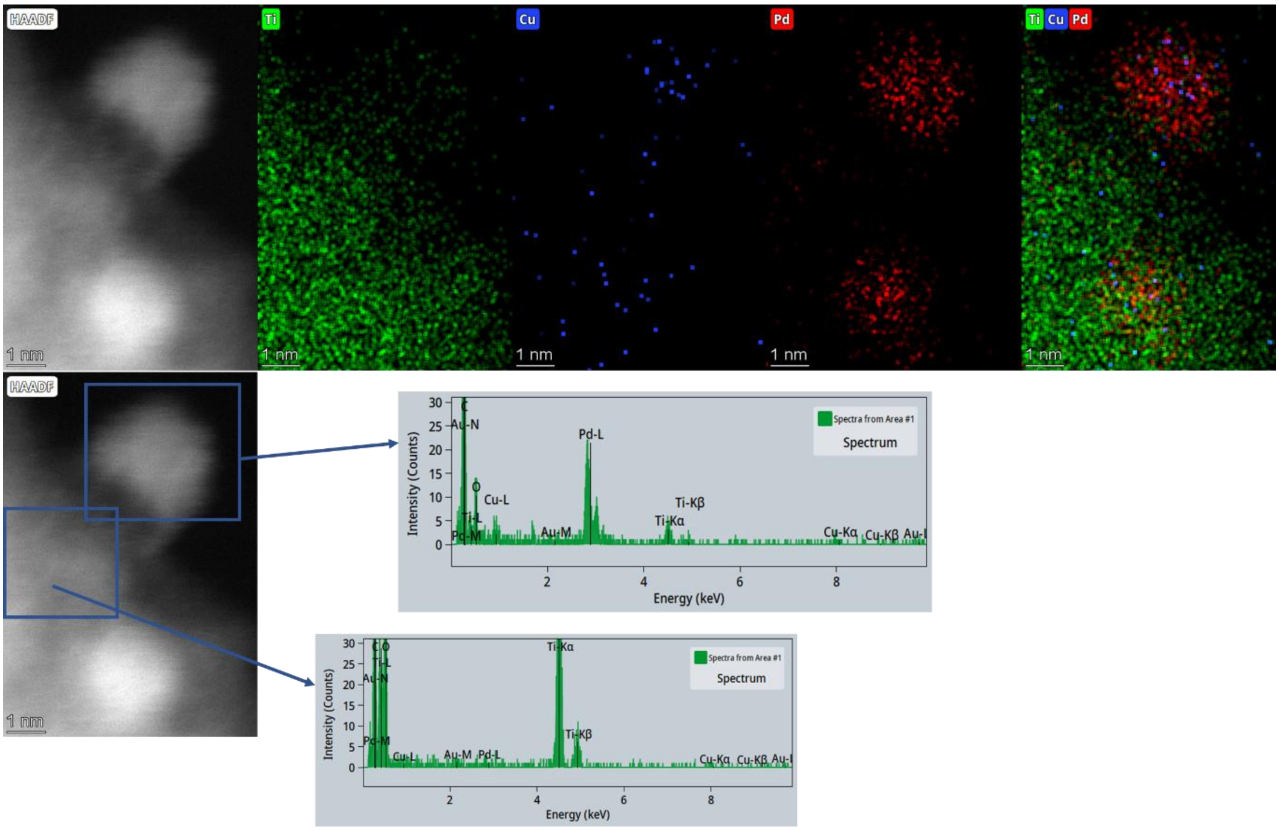

Supplementary Fig. 10 Side view of HAADF-STEM image and EDX element mapping of selected area (element mapping of the PdCu nanoalloy and the support).

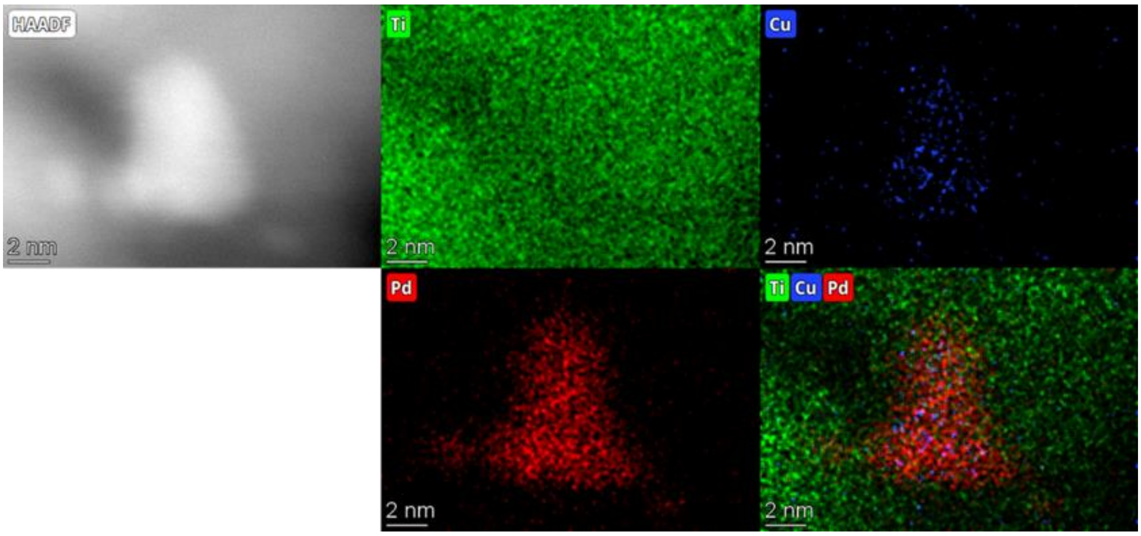

Supplementary Fig. 11 Top view of HAADF-STEM image and EDX element mapping of PdCu/TiO_2 after reactions.

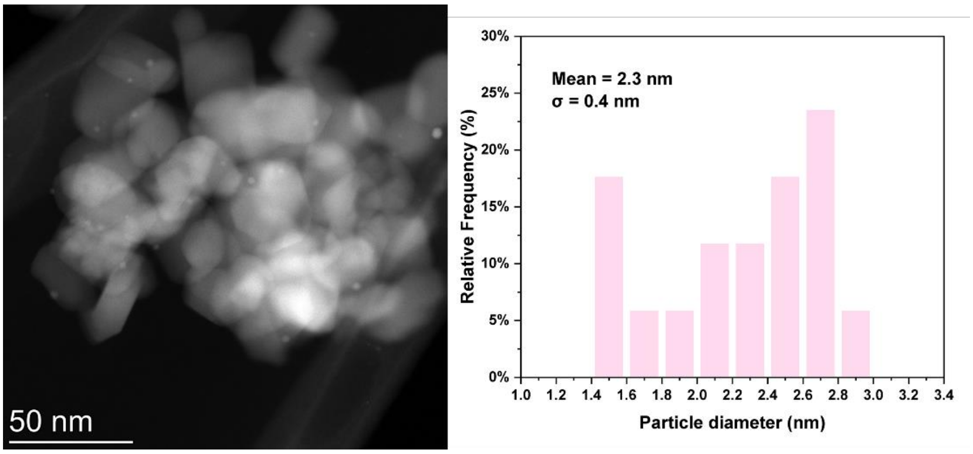

Supplementary Fig. 12 Representative STEM image and particle size distributions of PdCu alloy over PdCu/TiO_2 after reactions.

a.

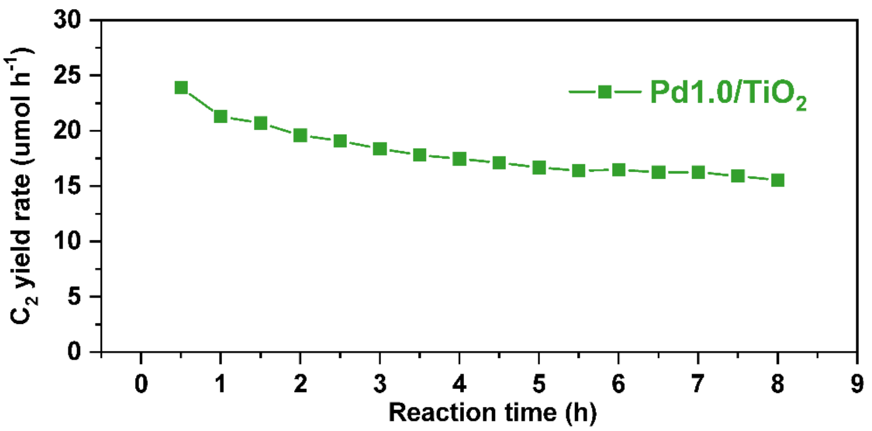

b.

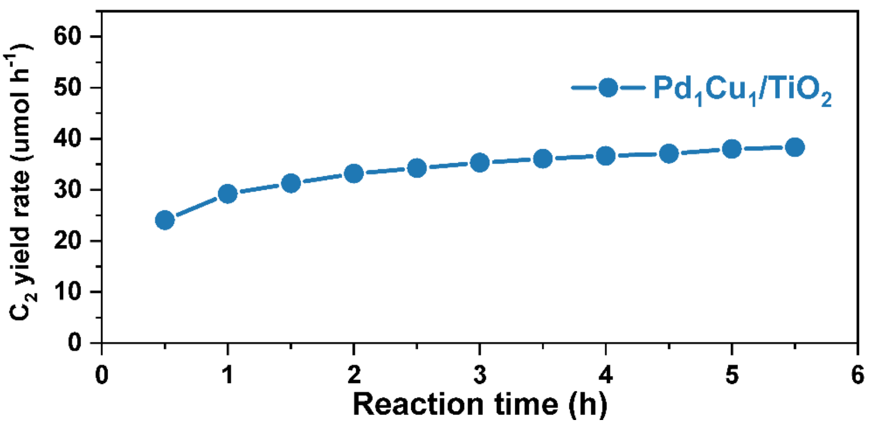

Supplementary Fig. 13 The trend of C_2 yield rate of $Pd_{1.0}/TiO_2$ (a) and Pd_1Cu_1/TiO_2 (b) over time.

It is reasonable to see a higher oxidation degree of Cu in our system since PdCu is proved as a photohole acceptor later, which can be oxidised readily. It should be noted that the Pd_1Cu_1/TiO_2 presented here with larger loading amount of Pd and Cu than the optimised sample (10 times higher than the optimised sample $PdCu/TiO_2$). Some photoholes may be consumed to oxidise Cu partially at the beginning of the reaction when the loading amount is so high, leading to a short induction period (<1.5 hour).

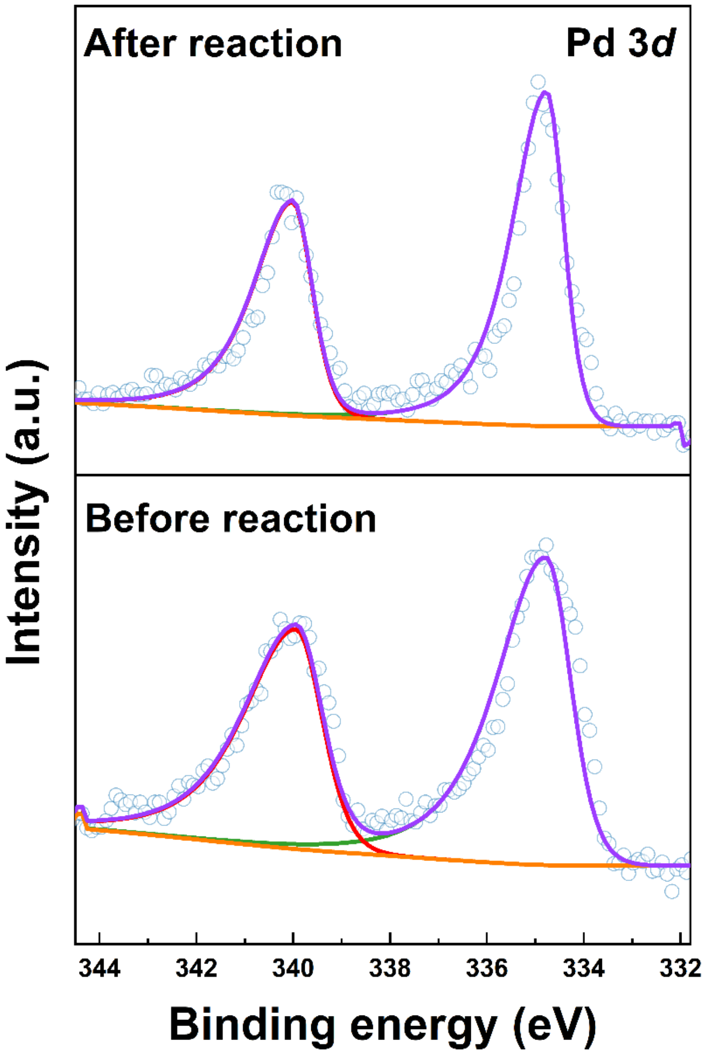

Supplementary Fig. 14 XPS spectra of Pd_1Cu_1/TiO_2 (Pd 3*d*) before and after reaction.

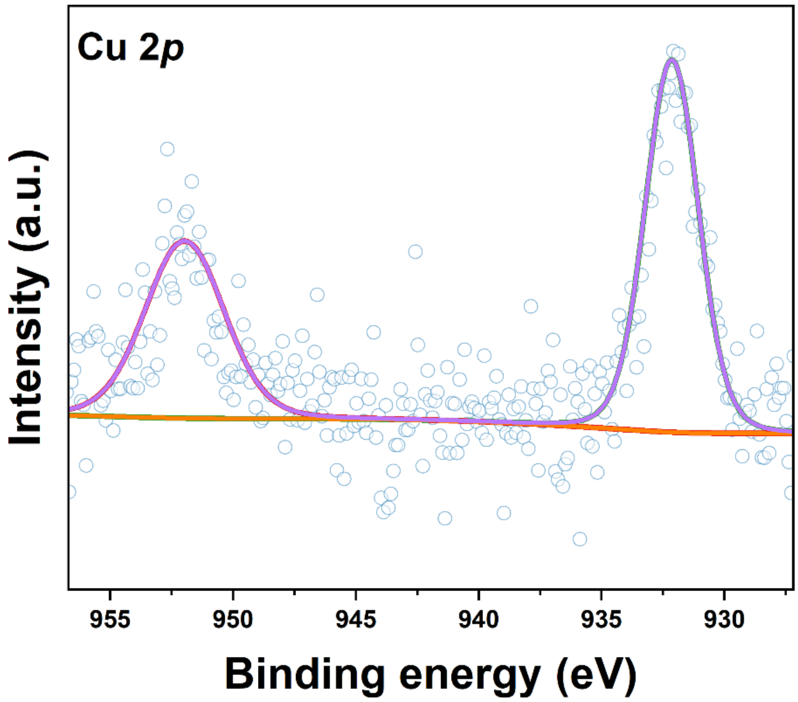

Supplementary Fig. 15 XPS spectra of Pd_1Cu_1/TiO_2 (Cu 2*p*) before reaction.

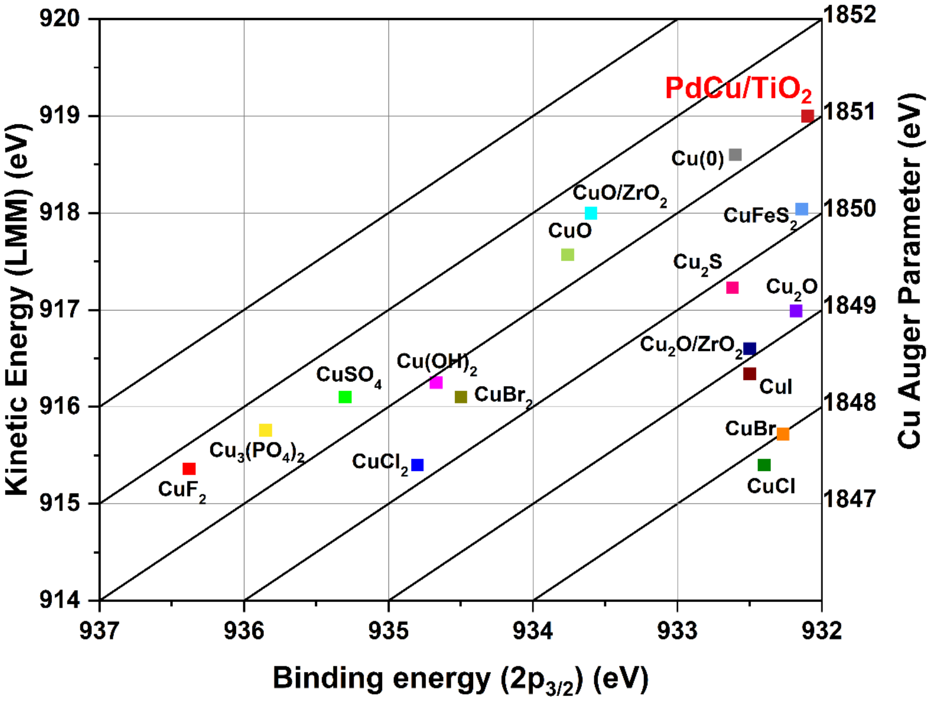

Supplementary Fig. 16 Wagner plot for Cu compounds from literatures (Supplementary Table 3) and $PdCu/TiO_2$ in this work.

Supplementary Table 3 Cu 2$p_{3/2}$ binding energy, Cu LMM kinetic energy, Auger parameter.

Compound	Cu 2$p_{3/2}$ E_b (eV)	Cu LMM E_k (eV)	Auger parameter α′(eV)
Cu(0)[17]	932.6	918.6	1851.2
Cu_2O[18]	932.18	916.99	1849.17
CuO[18]	933.76	917.57	1851.33
$Cu(OH)_2$[18]	934.67	916.25	1850.92
CuF_2[18]	936.38	915.36	1851.74
CuCl[19]	932.4	915.4	1847.8
$CuCl_2$[19]	934.8	915.4	1850.2
CuBr[18]	932.27	915.72	1848.00
$CuBr_2$[18]	934.50	916.10	1850.60
CuI[18]	932.50	916.34	1848.84
$Cu_3(PO_4)_2$[18]	935.85	915.76	1851.61
$CuSO_4$[19]	935.3	916.1	1851.4
Cu_2S[18]	932.62	917.23	1849.84
$CuFeS_2$[18]	932.14	918.04	1850.18
CuO/ZrO_2[20]	933.6	918	1851.6
Cu_2O/ZrO_2[20]	932.5	916.6	1849.1
$PdCu/TiO_2$	932.1	919.0	1851.1

Note: $\alpha' = E_b + E_k$.[21]

Wagner plot formed by binding energy versus the kinetic energy allow us to compare the binding energy (E_b) and modified Auger parameter (α′, known now as simply the Auger parameter) with the model systems, which gives more insight into the electronic states of the metal compounds in addition to the binding energy envelope[18,20–22]. Thus, the Cu 2$p_{3/2}$ binding energy and Cu LMM kinetic energy (E_k) data from literatures (Supplementary Table 3) were used to generate the Wagner plot as shown

in Supplementary Figure 16. A trend can be seen that compounds with a Cu(II) follow the line with similar Auger Parameter value at ca. 1851 eV. Notably, the position of our sample PdCu/TiO_2 is also close to this trend line, suggesting that Cu(II) species likely exists in the sample. In contrast, the compounds with only Cu(0) or Cu(I) exhibit a similar binding energy with greatly different Auger parameters. PdCu/TiO_2 also shows a similar binding energy to them and a closer Auger parameters to Cu(0) than to Cu(I). This result implies that Cu(0) or Cu(I) species may also exist in the sample PdCu/TiO_2 and the former has a higher probability. The combination of different technologies (e.g., EXAFS) to reveal the chemical state of Cu is necessary.

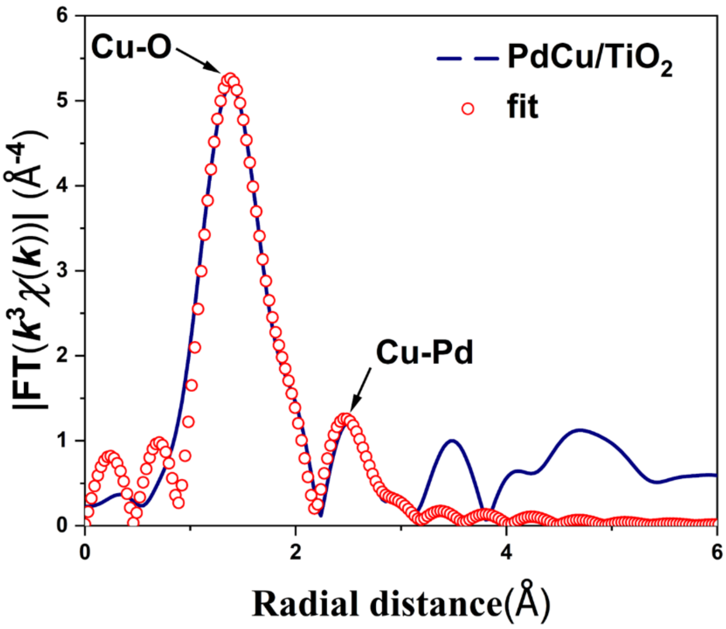

Supplementary Fig. 17 Fourier transformation magnitudes of k^3 – weighted EXAFS data and theoretical fits of Cu K-edge for $PdCu/TiO_2$.

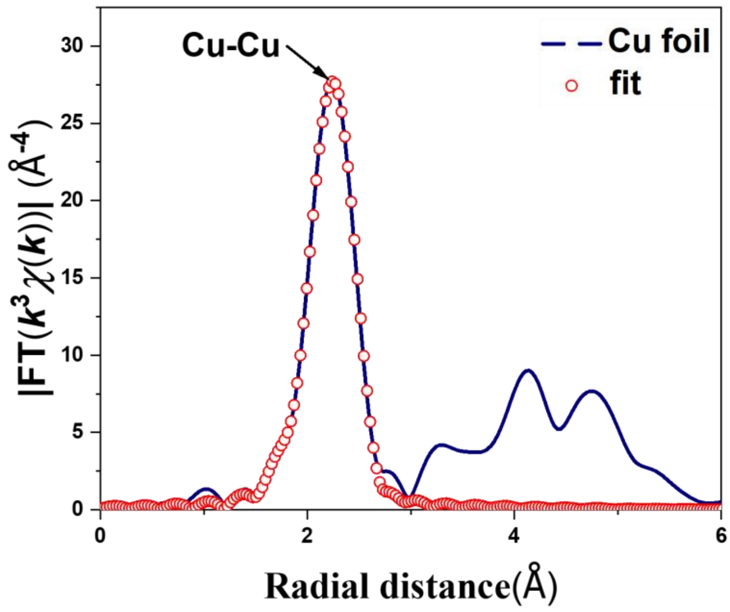

Supplementary Fig. 18 Fourier transformation magnitudes of k^3 – weighted EXAFS data and theoretical fits of Cu K-edge for Cu foil.

Supplementary Table 4 EXAFS fitting parameters at the Cu K-edge for various samples

Sample	Shell	CN^a	R(Å)^b	σ^2(Å^2)^c	ΔE_0(eV)^d	R factor
Cu foil	Cu-Cu	12*	2.542±0.003	0.0086±0.0003	4.4±0.4	0.0036
$PdCu/TiO_2$	Cu-O	2.6±0.4	1.872±0.026	0.0063±0.0046	4.2±3.1	0.0185
	Cu-Pd	1.5±0.2	2.758±0.010	0.0165±0.0080	-5.6±9.5	
$PdCu/TiO_2$ after reaction	Cu-O	4.4±0.4	2.021±0.018	0.0063±0.0046	0.7±2.1	0.0084
	Cu-Pd	0.8±0.3	2.767±0.024	0.0126±0.0013	8.2±4.1	

[a]*CN*, coordination number; [b]*R*, the distance to the neighboring atom; [c]σ^2, the Mean Square Relative Displacement (MSRD); [d]ΔE_0, inner potential correction; *R* factor suggests the well-fitting. S_0^2 was fixed to 0.901, according to the experimental EXAFS fit of Cu foil by fixing *CN* as the known crystallographic value. * This value was fixed based on the known structure of Cu. Fitting range: $3.0 \le k$ (/Å) ≤ 13.6 and $1.0 \le R$ (Å) ≤ 3.0 (Cu foil); $2.0 \le k$ (/Å) ≤ 10.0 and $1.0 \le R$ (Å) ≤ 3.0 (Sample Cu). A reasonable range of EXAFS fitting parameters: $0.700 < S_0^2 < 1.000$; $CN > 0$; $\sigma^2 > 0$ Å^2; $|\Delta E_0| < 10$ eV; R factor < 0.02.

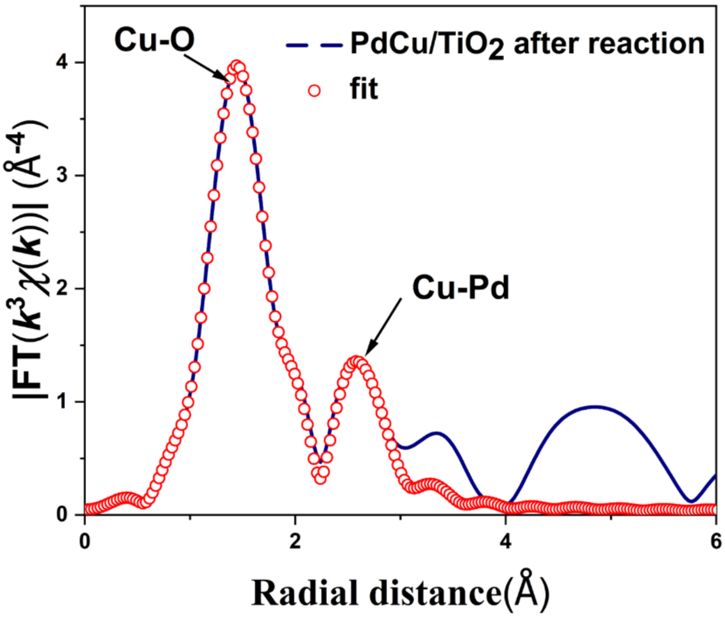

Supplementary Fig. 19 Fourier transformation magnitudes of k^3 – weighted EXAFS data and theoretical fits of Cu K-edge for $PdCu/TiO_2$ after the reaction.

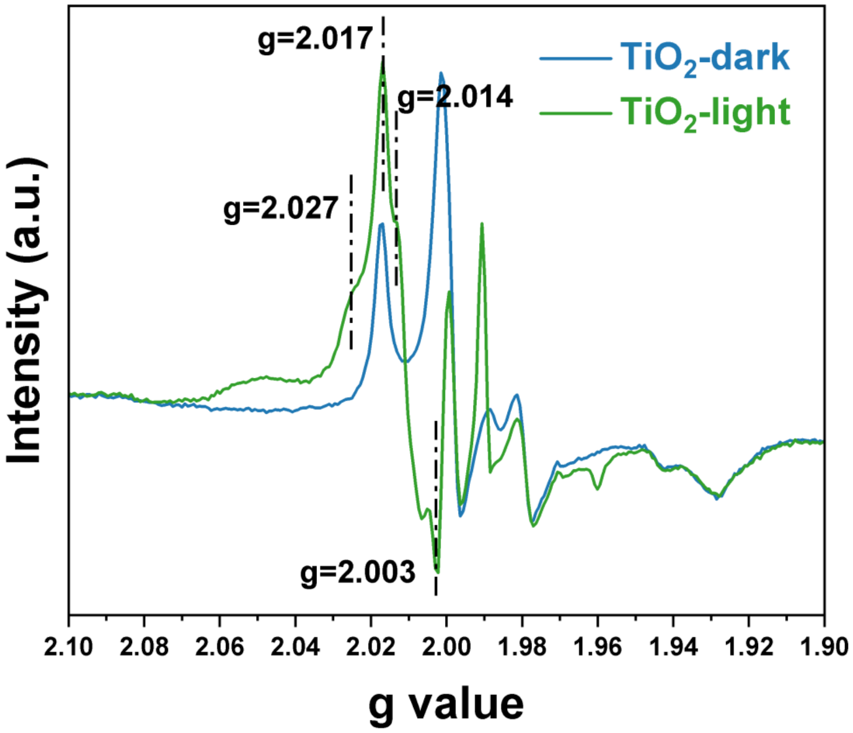

Supplementary Fig. 20 In-situ EPR spectra of TiO_2 under dark condition and light irradiation in argon atmosphere

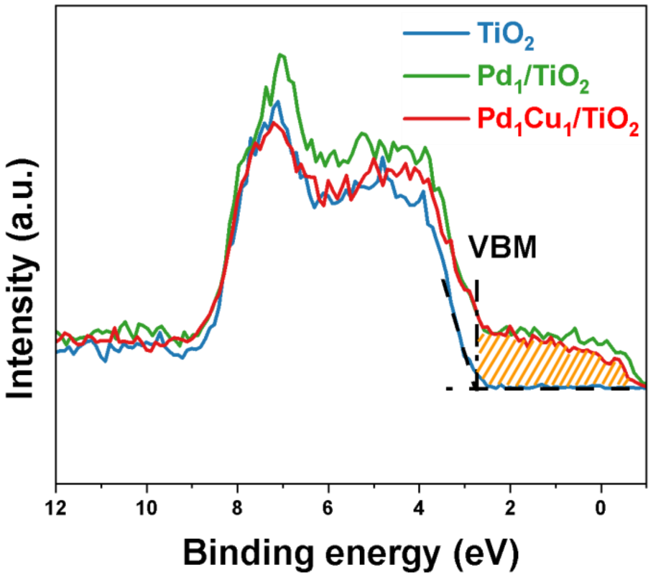

Supplementary Fig. 21 Valence-band XPS spectra of TiO_2, Pd_1/TiO_2, and Pd_1Cu_1/TiO_2.

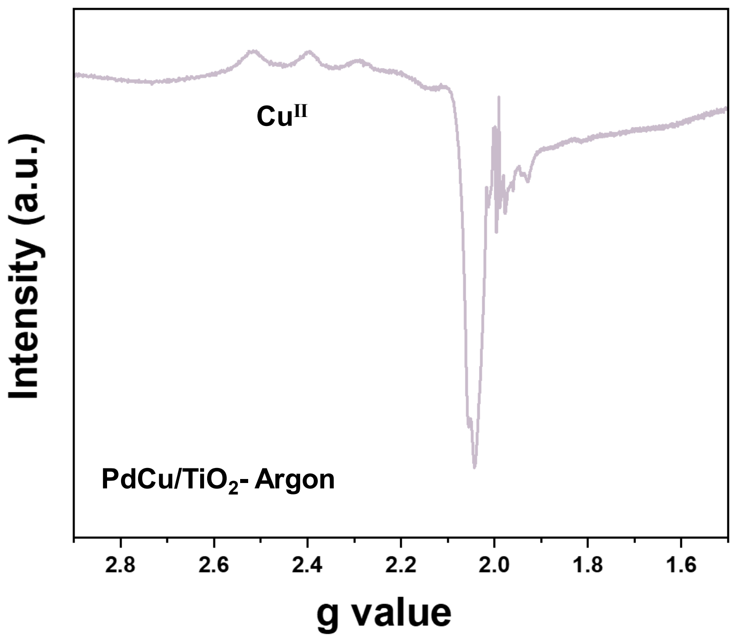

Supplementary Fig. 22 In-situ EPR spectra of $PdCu/TiO_2$ under light irradiation in argon atmosphere.

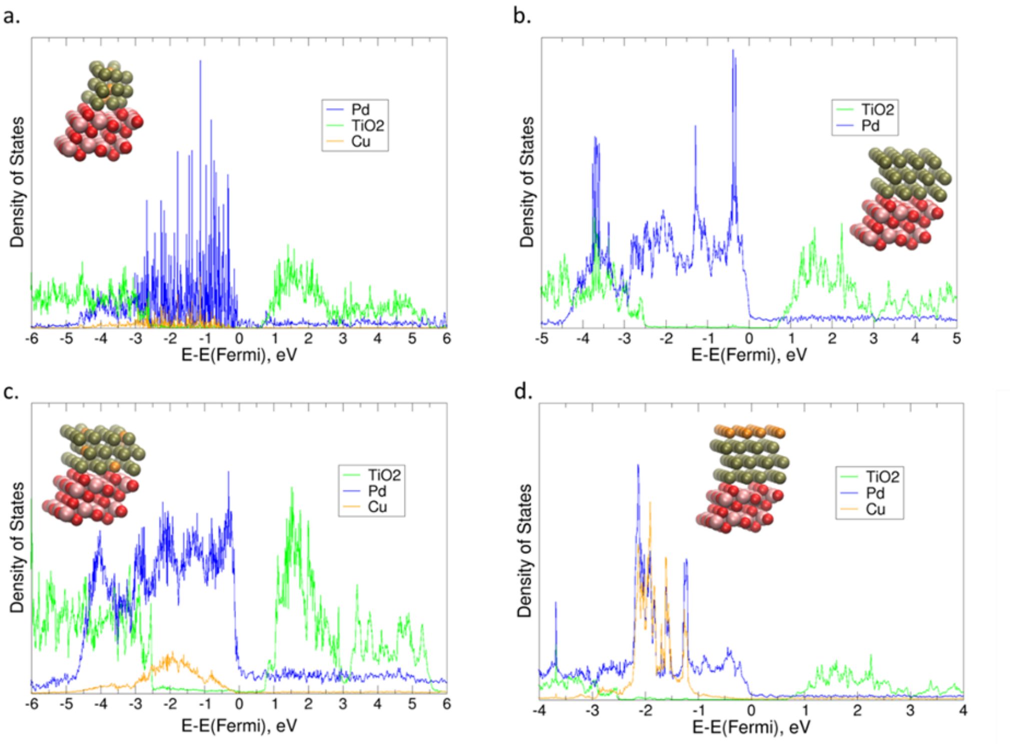

Supplementary Fig. 23 Calculated density of electronic states of different PdCu/TiO_2 models (a. TiO_2 with $Pd_{24}Cu_3$ nanoparticle; b. TiO_2 with 3-layer Pd; c. TiO_2 with 3-layer PdCu alloy; d. TiO_2 with 3-layer Pd and one layer Cu; red – O, pink – Ti, tan – Pd, orange – Cu).

Four different models were constructed to investigate the electronic states. The models (shown in Supplementary Fig. 23) contain a TiO_2 anatase (101)-oriented slab with various Pd or PdCu adsorbates: (a) a finite $Pd_{24}Cu_3$ nanoparticle adsorbed on TiO_2, (b) 3-layer Pd slab adsorbed on TiO_2, (c) 3-layer PdCu alloy (Pd:Cu ratio ≈ 9:1) adsorbed on TiO_2, (d) a 3-layer Pd slab topped with 1 layer Cu, adsorbed on TiO_2. In all examples, there are occupied Pd states and Cu states located at and above the valence band maximum of TiO_2, consistent with the observations in valence-band XPS measurements (Supplementary Fig. 21). It is favorable for the holes to fill states that are high energy, leading to the possibility of hole accumulation on metallic species. In other words, these metal-based occupied states can donate electrons to consume the photoholes at the valence band of TiO_2, which may form additional active sites in photocatalysis as reported previously.[23] The charge transfer to/from adsorbed metal to TiO_2 over these four models were also investigated, as shown in Supplementary Table 5.

Supplementary Table 5 Charge transfer from metal species to TiO_2

Metal species	$Pd_{24}Cu_3$ nanoparticle	3-layer Pd	3-layer PdCu alloy	3-layer Pd and one layer Cu
dQ on TiO_2	-0.31	-0.20	-0.16	-0.20
dQ on PdCu or Pd	0.30	0.20	0.16	0.20

Note: dQ indicates the difference between the calculated Mulliken charges on atoms and the formal nuclear charges. The negative value indicates gain of electron density while the positive value indicates loss of electron density.

Mulliken charges on all atoms were calculated and compared to formal charges on the neutral species, to find out the amount of charges transferred (dQ). Negative dQ indicates that the species gain electrons, while positive dQ means that the species lose electrons. The Mulliken charges data for all four systems show that the metal species in the combined TiO_2/Pd or PdCu systems indeed lose electrons, while TiO_2 gains electrons. This is consistent with the in-situ EPR and in-situ NEXAFS results above. Therefore, PdCu works as a photohole acceptor and the photohole could transfer from TiO_2 to PdCu.

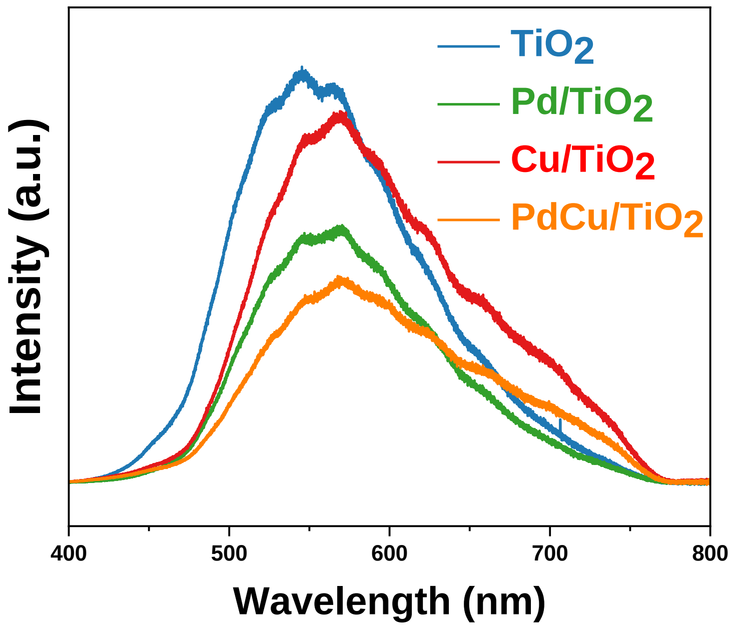

Supplementary Fig. 24 Photoluminescence spectra of TiO_2, Pd/TiO_2, Cu/TiO_2 and PdCu/TiO_2.

Although all four samples have similar light absorption based on UV-Vis DRS spectra (Fig. 2c), different intensities of photoluminescence were observed over four samples. Among them, pristine TiO_2 displays the highest PL intensity, suggesting the highest radiative recombination rates of photo-induced electrons and holes. After loading Pd or Cu, the PL intensity is reduced, and loading the nanoalloy dramatically reduces the PL intensity by 50%, likely due to effective enhancement of the charge transfer and separation in TiO_2.

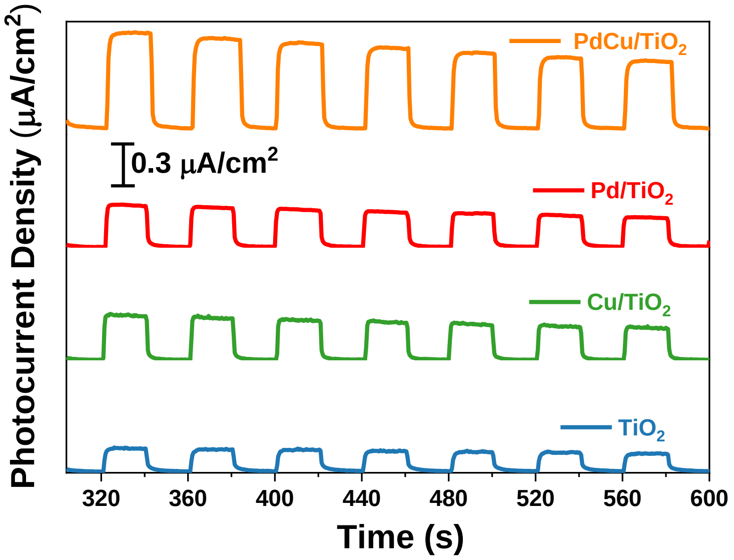

Supplementary Fig. 25 Photocurrent densities of TiO_2, Pd/TiO_2, Cu/TiO_2 and PdCu/TiO_2.

PdCu/TiO_2 also exhibits the highest photocurrent, more than 4 and 2 times higher than the pristine TiO_2 and single metal decorated TiO_2, respectively, which consolidates the efficient transfer of photo-induced carriers from TiO_2 to the nanoalloy.

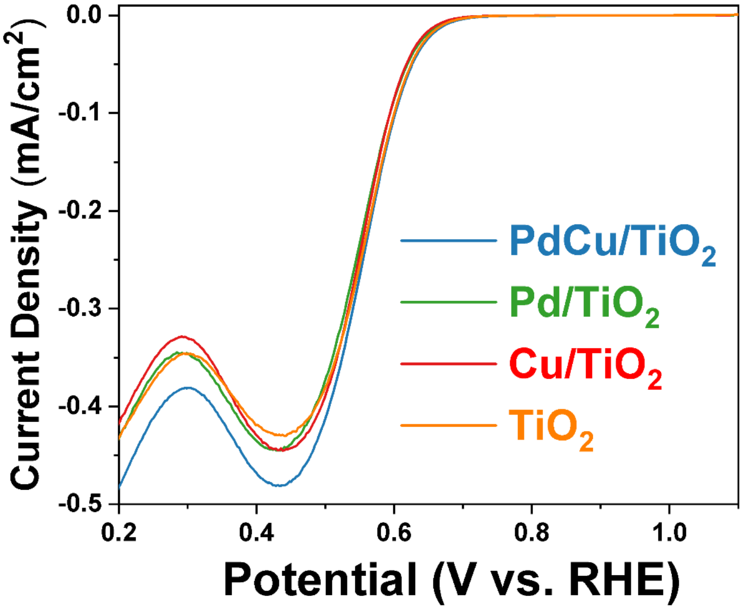

Supplementary Fig. 26 Electrochemical oxygen reduction spectra over TiO_2, Pd/TiO_2, Cu/TiO_2 and PdCu/TiO_2 in 0.1 M KOH electrolyte.

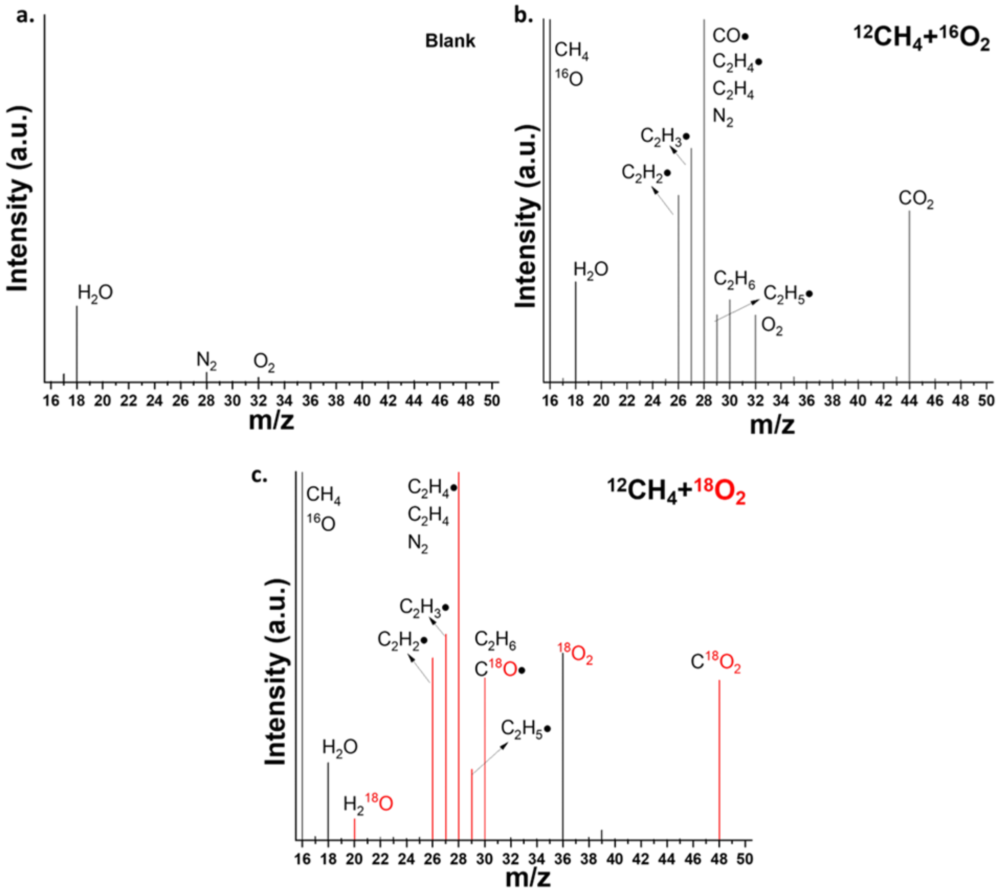

Supplementary Fig. 27 Mass spectra of the background (a), mass spectra of the products using $^{12}CH_4$ + $^{16}O_2$ as the feed gas (b) and mass spectra of products using $^{12}CH_4$ + $^{18}O_2$ as the feed gas (c). (The peaks assigned to R· are molecular fragments of the main products. Reaction condition: 20 mg PdCu/TiO_2, CH_4: O_2 = 80: 1, total flow rate of 120 mL min^{-1}, 300W Xenon lamp irradiation.)

Note: the ion box of the MS cannot reach 100% vacuum condition by the vacuum pump and the air or moisture from the environment can somehow enter the ion box to give the signals of m/z = 18, 28, 32. If every factor (e.g., environment, working temperature) remains relatively stable, the intensity of the above signals would remain constant with little fluctuation. Then, such intensity can be regarded as the baseline. However, it still needs to be very careful when dealing with these signals if the intensity of detective components is not high enough compared with the background since the environment may change, e.g., humidity etc. Thus, it is highly recommended to obtain the background spectra first on the same date of sample measurement to avoid any misleading.

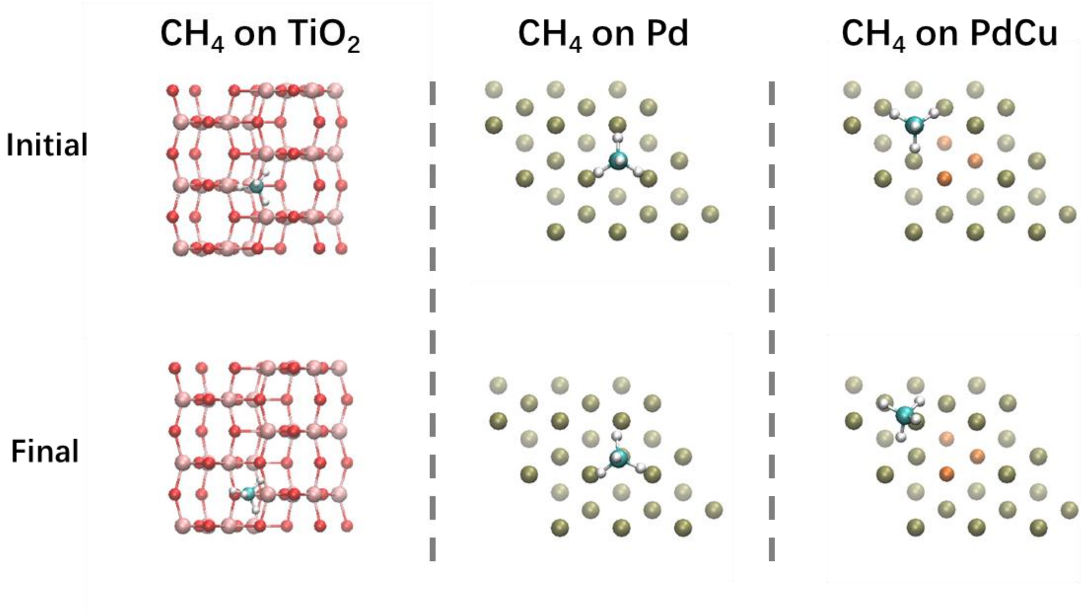

Supplementary Fig. 28 DFT calculated structures of methane adsorption on anatase, Pd and PdCu surfaces (red – O, pink – Ti, tan – Pd, orange – Cu, teal – C, white – H. Brighter colours of metal atoms denote atoms in the top (surface) layer).

A series of configurations for methane adsorption on the (101) surface of anatase TiO_2, (111) surface of Pd and (111) surface of PdCu were screened, and three representative initial structures and the corresponding final structures with lowest energies are displayed here.

Supplementary Table 6 DFT calculations results of methane adsorption on TiO_2, Pd and PdCu.

System	Adsorption energy (kJ/mol)	C-H bond distance (H down) (Å)	C-H bond distance (H down) (Å)	C-H bond distance (H down) (Å)	C-H bond distance (H up) (Å)
CH_4 on TiO_2	-22.54	1.101	1.101	1.099	1.099
CH_4 on Pd	-24.71	1.104	1.103	1.102	1.101
CH_4 on PdCu	-22.38	1.106	1.104	1.099	1.000

Supplementary Table 7 Activation energy of dissociation of C-H bond of adsorbed CH_4

Surface	Activation barrier (kJ/mol)
Gas phase	473
TiO_2	269.5
Pd	77.7
PdCu	77.7 (near Pd) or 119.8 (near Cu)

Supplementary Table 8 Activation energy for ethane formation on different catalysts.

Reaction	Activation energies (kJ/mol)		
	On Pd	On PdCu	On TiO_2
$CH_3\cdot + CH_3\cdot \rightarrow C_2H_6$	175.5	175.6	124.9
$CH_4 + CH_3\cdot \rightarrow C_2H_6 + H\cdot$	355.4	304.5	Not converged
$CH_4 + CH_4 \rightarrow C_2H_6 + 2H\cdot$	439.2	251.2	226.7

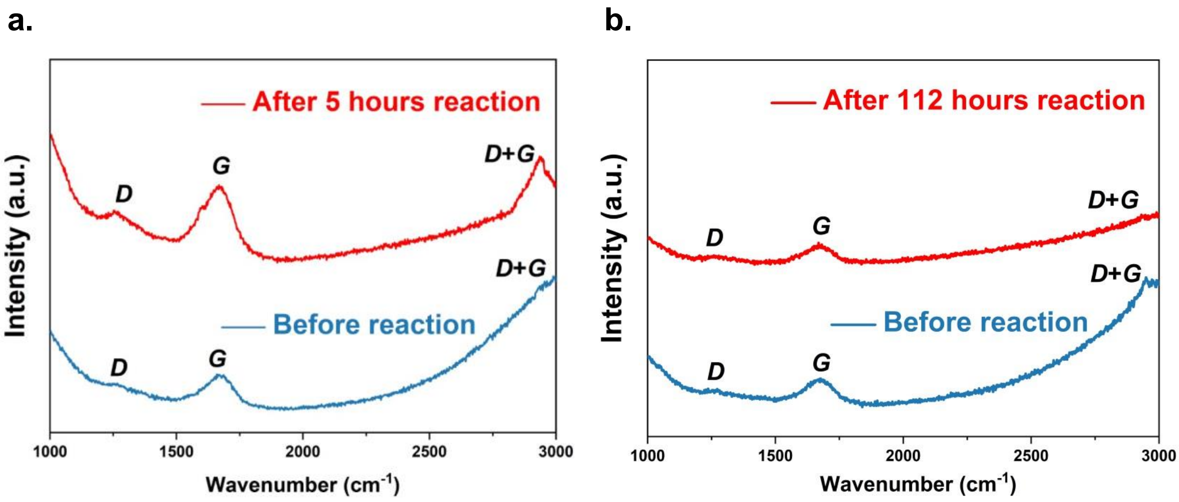

Supplementary Fig. 29 a. Raman spectra of Pd/TiO_2 before and after 5 hours reaction in the range of 1000-3000 cm^{-1}. b. Raman spectra of PdCu/TiO_2 before and after 112 hours reaction in the range of 1000-3000 cm^{-1}.

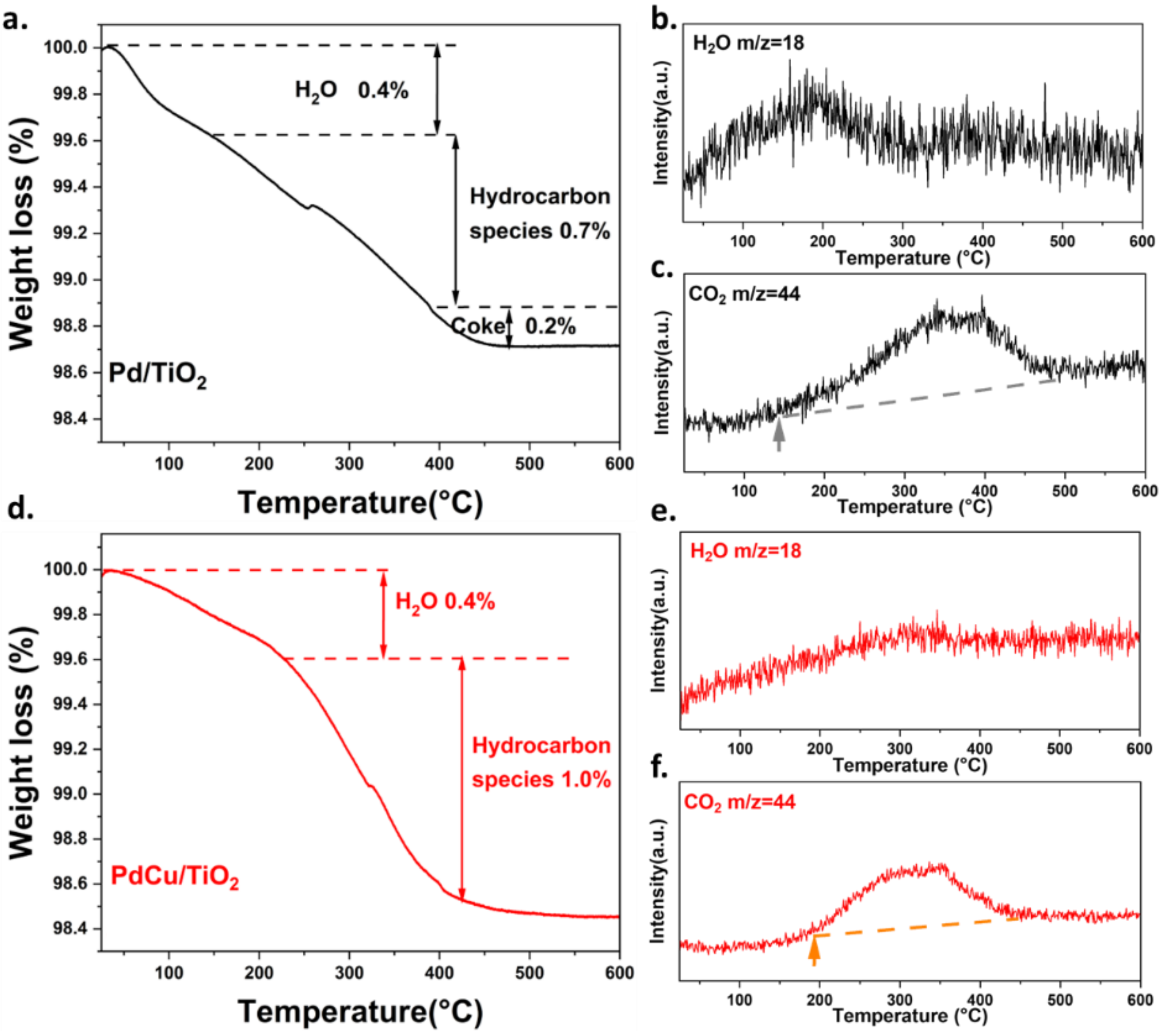

Supplementary Fig. 30 TGA-MS spectra of Pd/TiO_2 after 10 hours reaction and PdCu/TiO_2 after 112 hours reaction (a. TGA spectra, b. MS spectrum of H_2O, c. MS spectrum of CO_2 over spent Pd/TiO_2; d. TGA spectra, e. MS spectrum of H_2O, f. MS spectrum of CO_2 over spent PdCu/TiO_2)

As shown in Supplementary Fig. 30a, three different species can be observed over Pd/TiO_2 after 10-hour reaction, corresponding to the H_2O (0.4%, between 50 °C and 150 °C), hydrocarbon species (0.7%, between ca. 150 °C and ca. 350 °C) and coke species (0.2%, between ca. 350 °C and ca. 500 °C). The attribution is based on the MS spectrum (Supplementary Fig. 30b, c), in which the signal for H_2O starts from 50 to ca. 300 °C and the signal for CO_2 start from ca. 150 °C to ca. 500 °C, peaking at around 350°C and 400 °C. The overlapped region between H_2O and CO_2 (peaking at 350°C) is considered as hydrocarbon species, probably due to the intermediates on the catalyst when the reaction was forced to stop. While the appearance of CO_2 (peaking at 400°C) without the noticeable formation of water is likely to be coke

species. Compared with Pd/TiO_2, the CO_2 MS signal over PdCu/TiO_2 starts from ca. 200 °C and peaks spanning from 300 °C to 350 °C (Supplementary Fig. 30f). The 50 °C lower of carbon oxidation peak temperature on PdCu/TiO_2 than on Pd/TiO_2 further suggests the existence of a more stubborn coke species on the latter (Supplementary Fig. 30c, f). Accordingly, the intensity of H_2O MS signal over PdCu/TiO_2 starts from ca. 50 °C and can increase up to ca. 400 °C, overlapped with the majority of CO_2 MS signal (from ca. 200 °C to ca. 400 °C), as shown in Supplementary Fig. 30e, f. Therefore, the stubborn coking is not an issue over PdCu/TiO_2. The solid coke species on Pd/TiO_2 likely cover the active sites to deactivate Pd/TiO_2, which is due to the deep dehydrogenation of CH_4 to C species. In comparison, the introduction of Cu can promote the desorption of as-formed products, thus avoiding the consecutive further reaction to form coke.

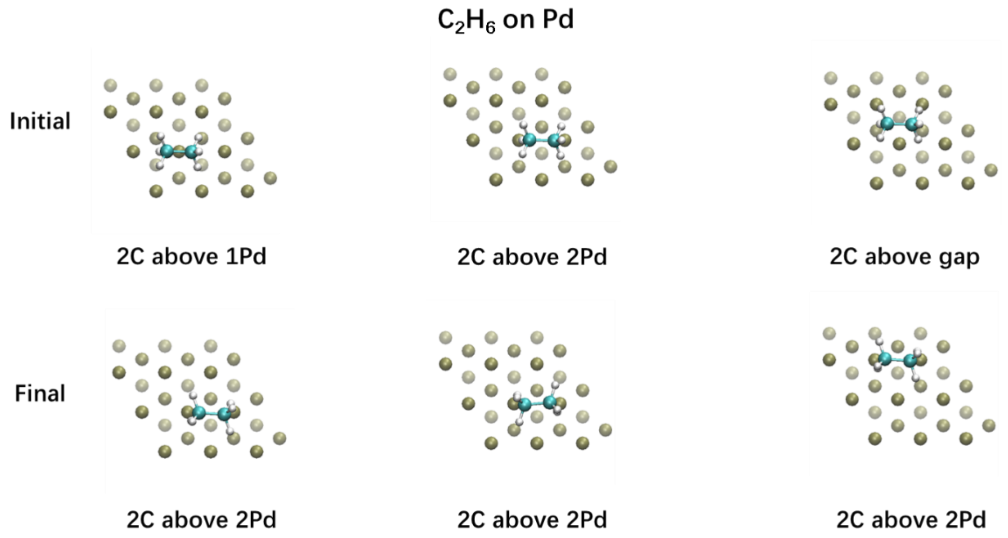

Supplementary Fig. 31 DFT calculation of ethane adsorption on Pd(111) surface. (Colours: tan – Pd, teal – C, white – H. Brighter colours of metal atoms denote atoms in the top (surface) layer)

Several initial positions of ethane on Pd (111) were investigated and final structures always end with two carbon atoms above two Pd atoms. The adsorption energies are also the same, thus one representative final structure with two carbons above two Pd atoms is displayed in Supplementary Fig. 32 to compare with that on PdCu.

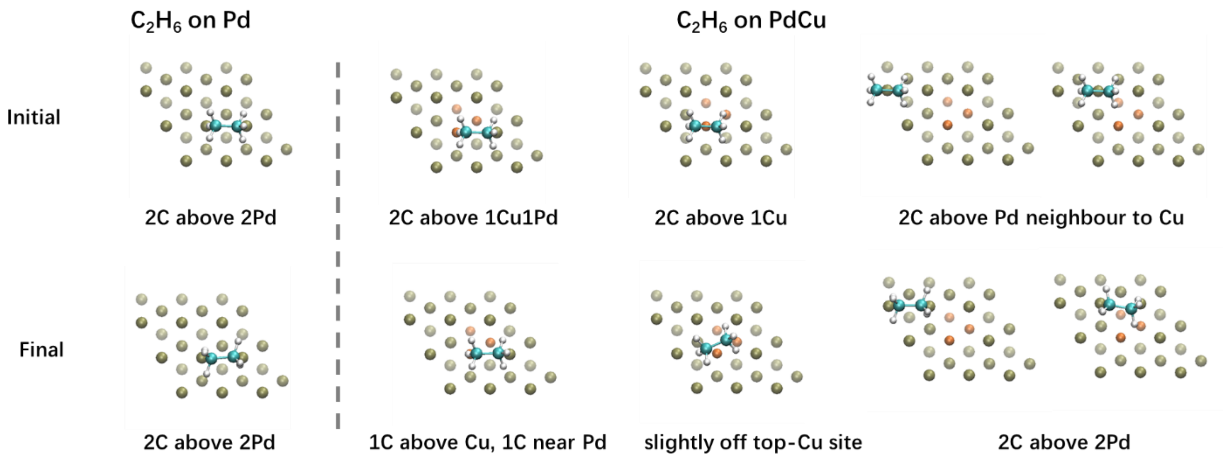

Supplementary Fig. 32 DFT calculated structures of ethane adsorption on Pd and PdCu surfaces. (tan – Pd, orange – Cu, teal – C, white – H. Brighter colours of metal atoms denote atoms in the top (surface) layer)

Representative structures of ethane adsorbed on a model alloy where one Pd atom per layer was replaced with one Cu atom were studied: a structure with one carbon atom of ethane above a Pd atom and the other carbon above a Cu atom, a structure with both carbons of ethane above one Cu, and two structures with two carbons above two Pd atoms neighbour to Cu.

Supplementary Table 9 DFT calculations results of ethane adsorption on Pd and PdCu.

System	Final structures	Adsorption energy (kJ/mol)
C_2H_6 on Pd	2 carbon above 2 Pd	-40.61
C_2H_6 on PdCu	1 carbon above Pd and 1 carbon above 1 Cu (direct involvement of Cu)	-36.80
C_2H_6 on PdCu	2 carbon above 1 Cu	-36.27
C_2H_6 on PdCu	2 carbon above 2 Pd neighboured to one Cu atom (indirect involvement of Cu)	-39.80 or -40.42 (depending on the distance of Pd to Cu)

Supplementary Table 10 DFT calculation results of oxygen vacancy creation by different species desorption

**Desorbing species**	**E (eV)**
O	9.37*
·OH	6.71
$\cdot OCH_3$	6.33
H_2O	2.33
CH_3OH	2.75

*The energy cost of desorbing O to create an oxygen vacancy is larger than the literature value[24], possibly due to the use of the DFT PBE functional in our work, while ref. 17 used a hybrid functional HSE06. However, even if the energy costs are overestimated, the trends in these energies are correct.

Supplementary Table 11 DFT calculation results of filling oxygen vacancy with O_2

**Species to fill the vacancy**	**E (eV)**
O_2	-5.81

Supplementary Table 12 DFT calculation results of dissociation of O_2 on the anatase surface

**System**	**E (eV)**
O_2 gas	5.21
O_2 on TiO_2 surface without vacancy	4.15
O_2 on TiO_2 surface with vacancy	1.17

When the methane molecule is activated by photoholes (e.g., Ti^{4+}-$O^{\cdot -}$-Ti^{4}, Ti^{4+}-O^{2-}-Ti^{4+}-$O^{\cdot -}$) on TiO_2,[25–27] the C-H bond of methane can be abstracted to form protons and methyl radicals. Therefore, we considered protons and methyl radicals as the most possible intermediates that may combine with the lattice oxygen in TiO_2 and their desorption energy to form oxygen vacancy (Supplementary table 10). We found that the easiest way to create an oxygen vacancy is when two H are simultaneously

adsorbed to surface O, which then can desorb by forming a H_2O and leaving an oxygen vacancy. Similar process has been observed in the activation of methane molecules in anaerobic system, e.g., non-oxidative coupling of methane.[1,28] The catalyst could be re-activated by either water washing or air treatment. This also indicates that the oxygen vacancy can be easily filled in the presence of oxygen source. As for the -OCH_3, it requires high energy to desorb, thus competing processes of consecutive dehydrogenation of OCH_3 to form adsorbed CH_2O and CHO and even CO_2 can happen on the surface of TiO_2, leading to formation of by-products other than C_2H_6.[29] Note that our reaction is oxidative coupling of methane (OCM) and oxygen was introduced with CH_4 during the reaction, therefore the oxygen vacancy can be replenished after formation. Thus, we have calculated the energy needed to fill the oxygen vacancy with O_2 (Supplementary table 11). The negative energy (-5.81 eV) indicates that the replenishing lattice oxygen vacancy by oxygen gas is a very favourable process.

We investigated the dissociation of O_2 on the surface with oxygen vacancy, as shown in Supplementary table 12. It is much easier to dissociate O_2 adsorbed at O-vacancy than on pure anatase TiO_2 surface. The above results suggest that the oxygen vacancy formed during the reaction can be in situ filled by oxygen gas. This is also consistent with the reported OCM process in the presence of oxygen[30] and different from the reported NOCM process in the absence of oxygen[1,28]. Our recent report using in situ Vis-NIR absorption spectroscopy technology also revealed that even trace amount of oxygen can effectively and efficiently react with the photoelectrons to generate superoxide radicals,[31] which is generally regarded as an active species to fill oxygen vacancy.[30] Therefore, the deactivation of catalyst by the loss of lattice oxygen is not the case in our aerobic reaction system, while the lattice oxygen may play a role in the formation of CO_2. Actually, the deactivation of catalyst can only be seen over Pd/TiO_2 (Supplementary Fig. 5), suggesting a different deactivation scheme revealed in the manuscript.

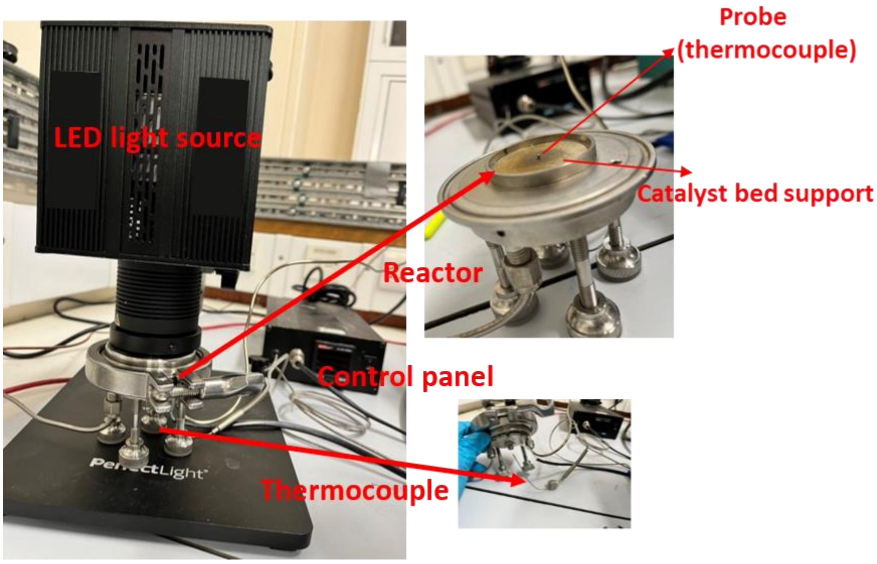

Supplementary Fig. 33 Physical photo of the reaction device.

Supplementary Reference

1. Jiang, W. *et al.* Pd-Modified ZnO–Au Enabling Alkoxy Intermediates Formation and Dehydrogenation for Photocatalytic Conversion of Methane to Ethylene. *J. Am. Chem. Soc.* **143**, 269–278 (2021).

2. Meng, L. *et al.* Gold plasmon-induced photocatalytic dehydrogenative coupling of methane to ethane on polar oxide surfaces. *Energy Environ. Sci.* **11**, 294–298 (2018).

3. Yuliati, L., Hattori, T., Itoh, H. & Yoshida, H. Photocatalytic nonoxidative coupling of methane on gallium oxide and silica-supported gallium oxide. *J. Catal.* **257**, 396–402 (2008).

4. Yuliati, L., Hattori, T. & Yoshida, H. Highly dispersed magnesium oxide species on silica as photoactive sites for photoinduced direct methane coupling and photoluminescence. *Phys. Chem. Chem. Phys.* **7**, 195 (2005).

5. Yuliati, L., Hamajima, T., Hattori, T. & Yoshida, H. Nonoxidative coupling of methane over supported ceria photocatalysts. *J. Phys. Chem. C* **112**, 7223–7232 (2008).

6. Ishimaru, M., Amano, F., Akamoto, C. & Yamazoe, S. Methane coupling and hydrogen evolution induced by palladium-loaded gallium oxide photocatalysts in the presence of water vapor. *J. Catal.* **397**, 192–200 (2021).

7. Yu, X. *et al.* Stoichiometric methane conversion to ethane using photochemical looping at ambient temperature. *Nat. Energy* **5**, 511–519 (2020).

8. Li, X., Xie, J., Rao, H., Wang, C. & Tang, J. Platinum- and CuOx -Decorated TiO2 Photocatalyst for Oxidative Coupling of Methane to C2 Hydrocarbons in a Flow Reactor. *Angew. Chemie Int. Ed.* **59**, 19702–19707 (2020).

9. Wu, S. *et al.* Ga-Doped and Pt-Loaded Porous TiO2 –SiO2 for Photocatalytic Nonoxidative Coupling of Methane. *J. Am. Chem. Soc.* **141**, 6592–6600 (2019).

10. Lang, J., Ma, Y., Wu, X., Jiang, Y. & Hu, Y. H. Highly efficient light-driven methane coupling under ambient conditions based on an integrated design of a photocatalytic system. *Green Chem.* **22**, 4669–4675 (2020).

11. Yuliati, L., Itoh, H. & Yoshida, H. Preparation of isolated highly dispersed titanium oxides on silica by sol-gel method for photocatalytic non-oxidative direct methane coupling. *Stud. Surf. Sci. Catal.* **162**, 961–968 (2006).

12. Kato, Y., Matsushita, N., Yoshida, H. & Hattori, T. Highly active silica-alumina-titania catalyst for photoinduced non-oxidative methane coupling. *Catal. Commun.* **3**, 99–103 (2002).

13. Kato, Y., Yoshida, H., Satsuma, A. & Hattori, T. Photoinduced non-oxidative coupling of methane over H-zeolites around room temperature. *Microporous*

Mesoporous Mater. **51**, 223–231 (2002).

14. Yuliati, L., Tsubota, M., Satsuma, A., Itoh, H. & Yoshida, H. Photoactive sites on pure silica materials for nonoxidative direct methane coupling. *J. Catal.* **238**, 214–220 (2006).

15. Wang, G. *et al.* Light-Induced Nonoxidative Coupling of Methane Using Stable Solid Solutions. *Angew. Chemie - Int. Ed.* **60**, 20760–20764 (2021).

16. Souza, J. D., Souza, V. S. & Scholten, J. D. Synthesis of Hybrid Zinc-Based Materials from Ionic Liquids: A Novel Route to Prepare Active Zn Catalysts for the Photoactivation of Water and Methane. *ACS Sustain. Chem. Eng.* **7**, 8090–8098 (2019).

17. van der Heide, P. A. W. Multiplet splitting patterns exhibited by the first row transition metal oxides in X-ray photoelectron spectroscopy. *J. Electron Spectros. Relat. Phenomena* **164**, 8–18 (2008).

18. Biesinger, M. C. Advanced analysis of copper X-ray photoelectron spectra. *Surf. Interface Anal.* **49**, 1325–1334 (2017).

19. D'Halluin, M. *et al.* Graphite-supported ultra-small copper nanoparticles - Preparation, characterization and catalysis applications. *Carbon N. Y.* **93**, 974–983 (2015).

20. Espinós, J. P. *et al.* Interface Effects for Cu, CuO, and Cu2O Deposited on SiO2 and ZrO2 . XPS Determination of the Valence State of Copper in Cu/SiO2 and Cu/ZrO2 Catalysts. *J. Phys. Chem. B* **106**, 6921–6929 (2002).

21. Biesinger, M. C., Lau, L. W. M., Gerson, A. R. & Smart, R. S. C. The role of the Auger parameter in XPS studies of nickel metal, halides and oxides. *Phys. Chem. Chem. Phys.* **14**, 2434–2442 (2012).

22. Barranco, A., Yubero, F., Espinós, J. P. & González-Elipe, A. R. The chemical state vector: A new concept for the characterization of oxide interfaces. *Surf. Interface Anal.* **31**, 761–767 (2001).

23. Jin, C. *et al.* Effects of single metal atom (Pt, Pd, Rh and Ru) adsorption on the photocatalytic properties of anatase TiO2. *Appl. Surf. Sci.* **426**, 639–646 (2017).

24. Li, H., Guo, Y. & Robertson, J. Calculation of TiO2 Surface and Subsurface Oxygen Vacancy by the Screened Exchange Functional. *J. Phys. Chem. C* **119**, 18160–18166 (2015).

25. Shoji, S. *et al.* Photocatalytic uphill conversion of natural gas beyond the limitation of thermal reaction systems. *Nat. Catal.* **3**, 148–153 (2020).

26. Hirakawa, T., Nakaoka, Y., Nishino, J. & Nosaka, Y. Primary passages for various TiO2 photocatalysts studied by means of luminol chemiluminescent probe. *J. Phys. Chem. B* **103**, 4399–4403 (1999).

27. Zhu, S. *et al.* Efficient Photooxidation of Methane to Liquid Oxygenates over ZnO Nanosheets at Atmospheric Pressure and Near Room Temperature. *Nano Lett.* **21**, 4122–4128 (2021).

28. Zhang, W. *et al.* High-performance photocatalytic nonoxidative conversion of methane to ethane and hydrogen by heteroatoms-engineered TiO2. *Nat. Commun.* **13**, 2806 (2022).

29. Tao, F. F. *et al.* Understanding complete oxidation of methane on spinel oxides at a molecular level. *Nat. Commun.* **6**, 7798 (2015).

30. Song, S. *et al.* A selective Au-ZnO/TiO2 hybrid photocatalyst for oxidative coupling of methane to ethane with dioxygen. *Nat. Catal.* **4**, 1032–1042 (2021).

31. Miao, T. J. *et al.* In Situ Investigation of Charge Performance in Anatase TiO2 Powder for Methane Conversion by Vis–NIR Spectroscopy. *ACS Catal.* **11**, 8226–8238 (2021).
